# Supplementary material for: Two distinct subpopulations of human stem-like memory T cells exhibit complementary roles in self-renewal and clonal longevity
Source: PLoS Biol. 2025 Jun 20;23(6):e3003179. doi: 10.1371/journal.pbio.3003179 (PMC12251145; doi:10.1371/journal.pbio.3003179)
Supplement: S1 Text — Fig A. Ki67 varies significantly in an independent dataset Fig B. Representative Gating. Fig C. Phenotype of sorted cells following 7 days ex vivo culture. Fig D. CTV profile of sorted cells following 7 days ex vivo culture. Fig E. CD95hi TSCM cells exhibit increased functionality compared to both CD95int TSCM cells and TCM cells. Fig F. Phenotypic characterization of CD8+ memory subsets. Fig G. Ki67 expression after exclusion of PD1+ TIGIT+ cells. Fig H. Heat maps of the 70 most differentially expressed genes. Fig I. CD95hi cells are transcriptionally and translationally closer to TCM while CD95int more closely resemble TN. Fig J. Schematic of Mechanistic Model to Describe Monocyte Kinetics. Fig K. Best fits of the models to the saliva and monocyte data. (A). Fig L. Fit of Model A (independent homogeneous populations) to all data (labelling, telomere, YFV) simultaneously. Fig M. Fit of Model B (independent heterogeneous populations) to all data (labelling, telomere, YFV) simultaneously. Fig N. Fit of Model C (fork) to all data (labelling, telomere, YFV) simultaneously. Fig O. Fit of Model E (linear, CD95hi first) to all data (labelling, telomere, YFV) simultaneously. Fig P. CD45A and CCR7 expression of the CD95hi TSCM and CD95int TSCM population. Fig Q. Predictions of Model C (fork) overlaid (NOT fitted) onto a novel data set. Fig R. Predictions of Model E (linear Thi first) overlaid (NOT fitted) onto a novel data set. Fig S. Duplicate of Fig 2 showing unique identifier for each donor in response to request from reviewer. (PDF) [file pbio.3003179.s001.pdf]

S1 Text

(Supplementary Figures)

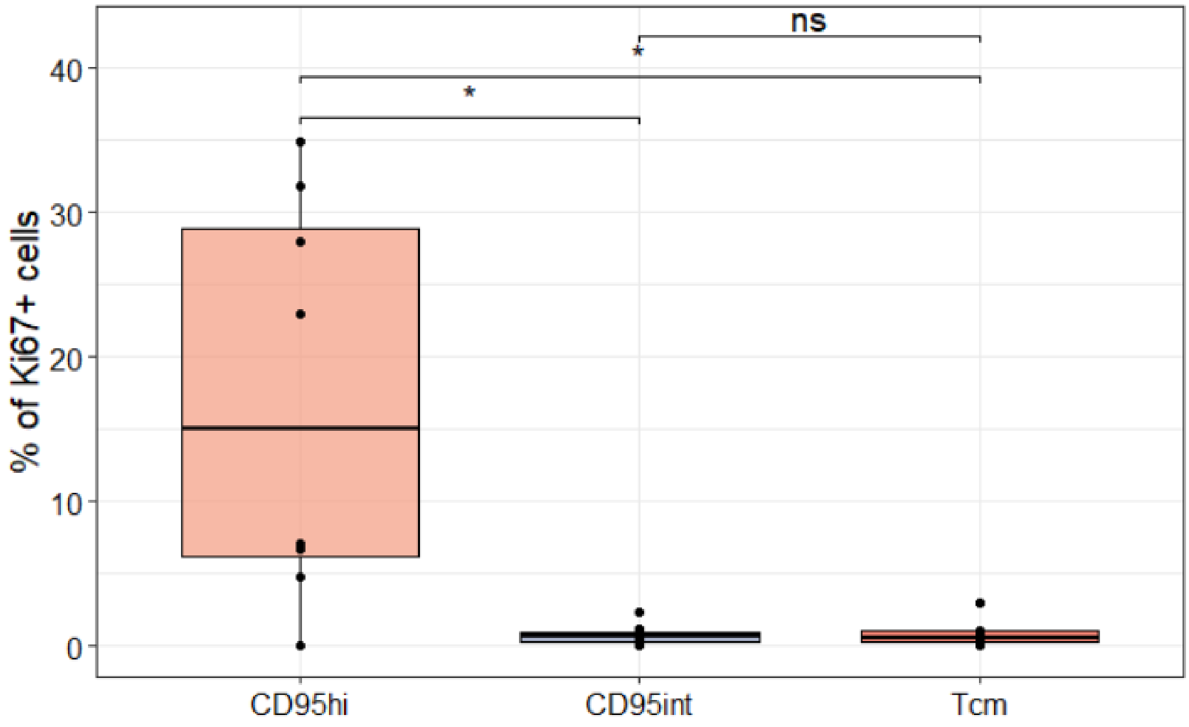

Figure A. Ki67 varies significantly in an independent dataset. To validate our finding that the frequency of Ki67 positivity was significantly higher in CD95hi T_{SCM} than CD95int T_{SCM} and T_{CM} we repeated our analysis in an independent cohort (Cohort 2, stained and analysed by independent collaborator). CD95hi v CD95int *P*=0.016, CD95hi v T_{CM} *P*=0.016, CD95int v T_{CM} P=0.46 N=8 two-tailed paired Wilcoxon test. The data underlying this figure can be found in **S1 Data.**

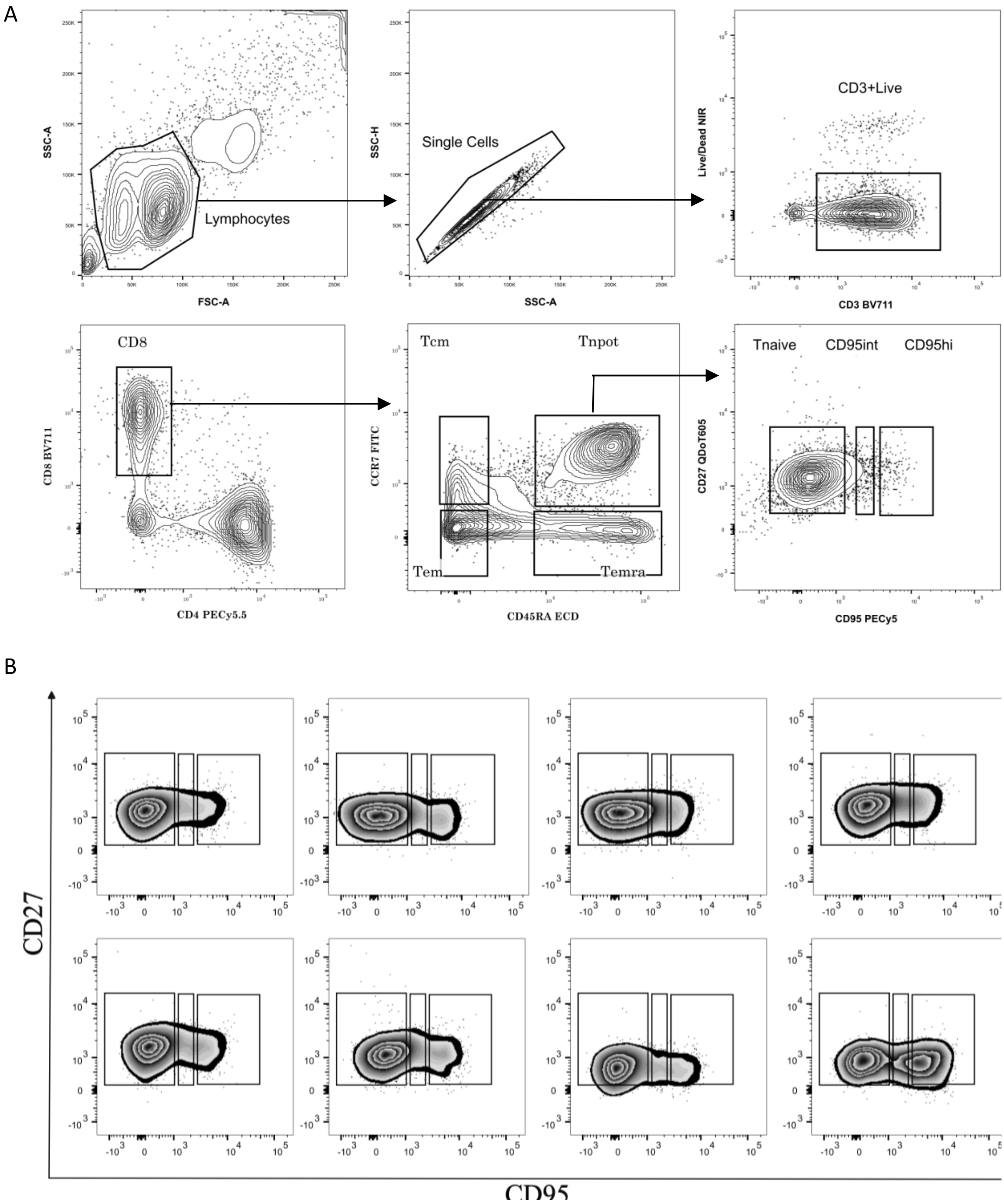
A
Lymphocytes
Single Cells
CD3+Live
SSC-A
FSC-A
SSC-H
Live/Dead NIR
CD3 BV711
CD8
CD8 BV711
CD4 PECy5.5
Tcm
Tnpot
Tem
Temra
CCR7 FITC
CD45RA ECD
Tnaive
CD95int
CD95hi
CD27 QDoT605
CD95 PECy5
B
CD27
CD95

C

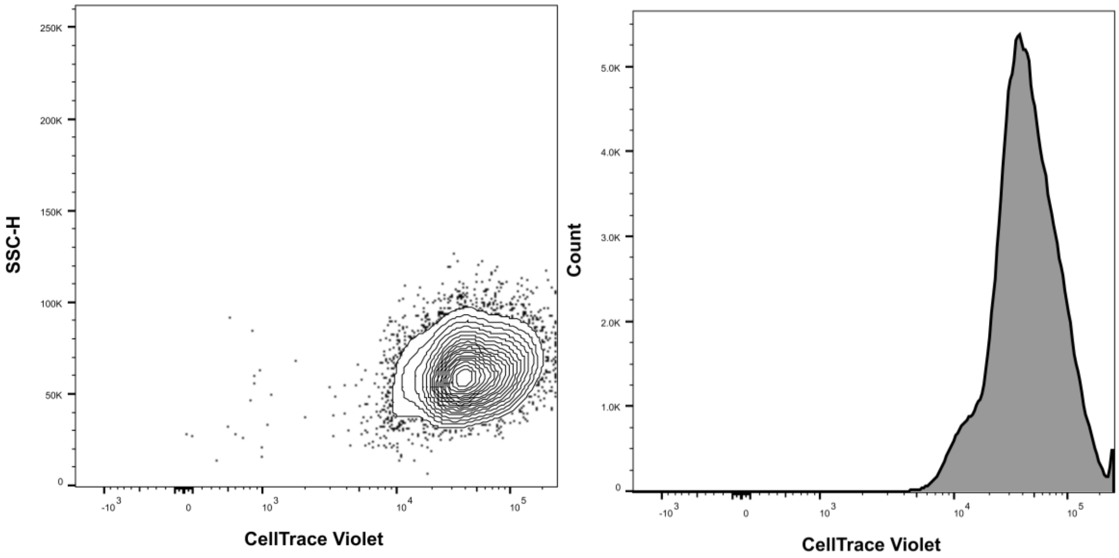

Figure B. Representative Gating. (A) Flow cytometry gating strategy for the isolation of $CD3^{+}CD8^{+}$ naive, central memory (T_{CM}), effector memory (T_{EM}), effector memory RA+ (T_{EMRA}), CD95hi T_{SCM} and CD95int T_{SCM} cells. Cells were defined as follows: T_N (naïve: $CD45RA^{+}CCR7^{+}CD95^{-}$), CD95int T_{SCM} (CD95 intermediate T stem cell like-memory: $CD45RA^{+}CCR7^{+}CD95^{INT}$), CD95hi T_{SCM} (CD95 high T stem cell like-memory: $CD45RA^{+}CCR7^{+}CD95^{HI}$), T_{CM} (central memory: $CD45RA^{-}CCR7^{+}$), T_{EM} (effector memory: $CD45RA^{-}CCR7^{-}$) and T_{EMRA} (CD45RA-expressing effector memory: $CD45RA^{+}CCR7^{-}$). **(B)** Gating strategy for the separation of CD95hi T_{SCM} and CD95int T_{SCM} from the $CCR7^{+}CD45RA^{+}$ “Tn pot” (potentially Tnaive) population of Panel A of Figure B shown for a further 8 individuals. **(C)** Cell Trace Violet (CTV) staining. See Figure D for CTV profiles following cell culture (and subsequent division). The data underlying this figure can be found in **S1 Data.**

A.

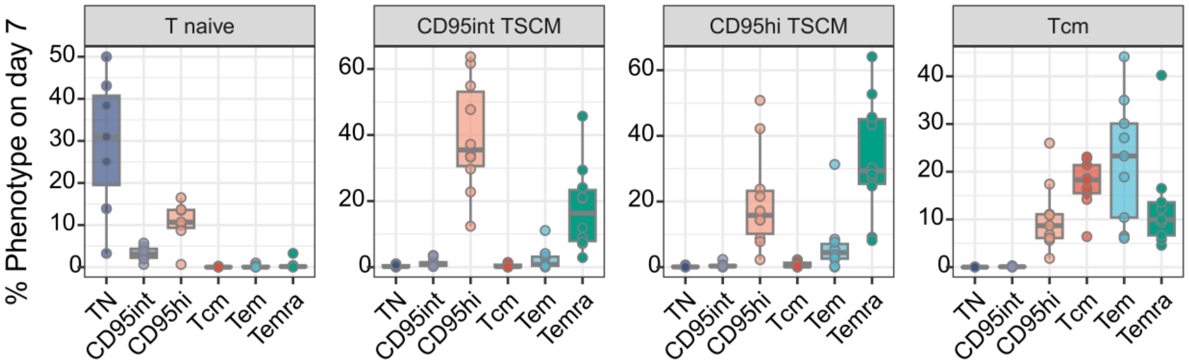

B

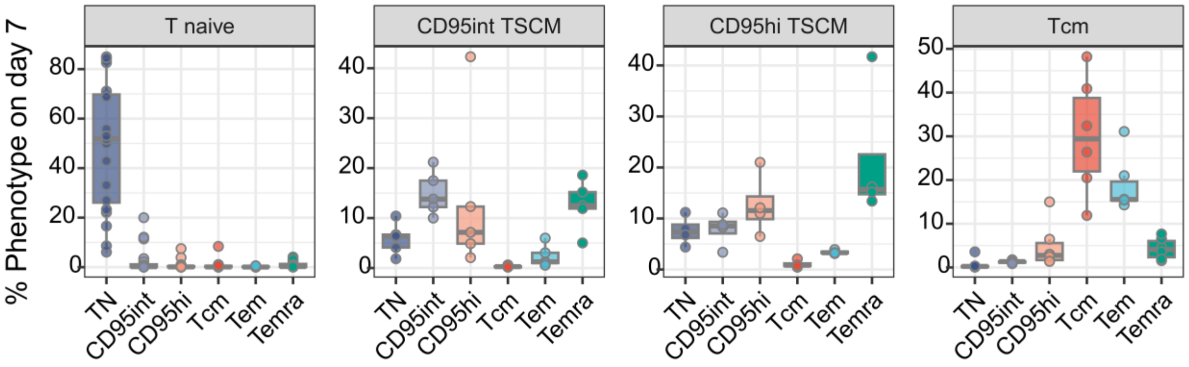

Figure C. Phenotype of sorted cells following 7 days *ex vivo* culture. $CD8^{+}$ Tnaive, CD95int T_{SCM}, CD95hi T_{SCM} and T_{CM} cells were isolated from PBMC by fluorescence-activated cell sorting. The phenotype of the starting (sorted) population is indicated in the grey bar above each panel. Cells were stimulated for 7d either with **(A)** IL-15 or **(B)** IL-2. Their phenotype at day 7 is plotted in the coloured bars. All donors were from cohort 1. Cells were defined as follows: T_N (naïve: $CD45RA^{+}CCR7^{+}CD95^{-}$), CD95int T_{SCM} (CD95 intermediate T stem cell like-memory: $CD45RA^{+}CCR7^{+}CD95^{INT}$), CD95hi T_{SCM} (CD95 high T stem cell like-memory: $CD45RA^{+}CCR7^{+}CD95^{HI}$), T_{CM} (central memory: $CD45RA^{-}CCR7^{+}$), T_{EM} (effector memory: $CD45RA^{-}CCR7^{-}$) and T_{EMRA} (CD45RA-expressing effector memory: $CD45RA^{+}CCR7^{-}$). Cells not falling into any of these gates at the time of the day 0 sort were discarded, similarly cells not falling into any of these gates at day 7 were not enumerated.

It can be seen that, when looking at all cells at day 7 (as opposed to only those cells that have divided when analysing self-renewal, **Fig 2A**) then CD95int T_{SCM} and CD95hi T_{SCM} differentiate in a similar manner to each other in response to homeostatic cytokines and show a different differentiation pattern to both Tnaive and T_{CM}.

These differentiation profiles also help to address the concern that CD95int T_{SCM} are simply T_{SCM} cells that are contaminated with Tnaive cells or that CD95hi T_{SCM} are simply T_{SCM} cells that are contaminated with T_{CM} cells. In response to IL-15, almost no CD95int T_{SCM} have a naïve phenotype and their differentiation profile is inconsistent with naïve cell contamination. In response to IL-2, very few CD95int T_{SCM} have a naïve phenotype (indeed similar levels are seen for cells sorted as CD95hi T_{SCM}) furthermore phenotypic reversion (memory cells acquiring a

naïve phenotype) has been previously documented [1, 2] so again there is little evidence that CD95int T_{SCM} are contaminated with Tnaive cells, and if they are, contamination of the CD95hi T_{SCM} population is at a comparable level. Similarly, for CD95hi T_{SCM}, in response to both IL-15 and IL-2, almost no cells have a T_{CM} phenotype and their differentiation profile in response to both cytokines is inconsistent with T_{CM} contamination.

The data underlying this figure can be found in **S1 Data.**

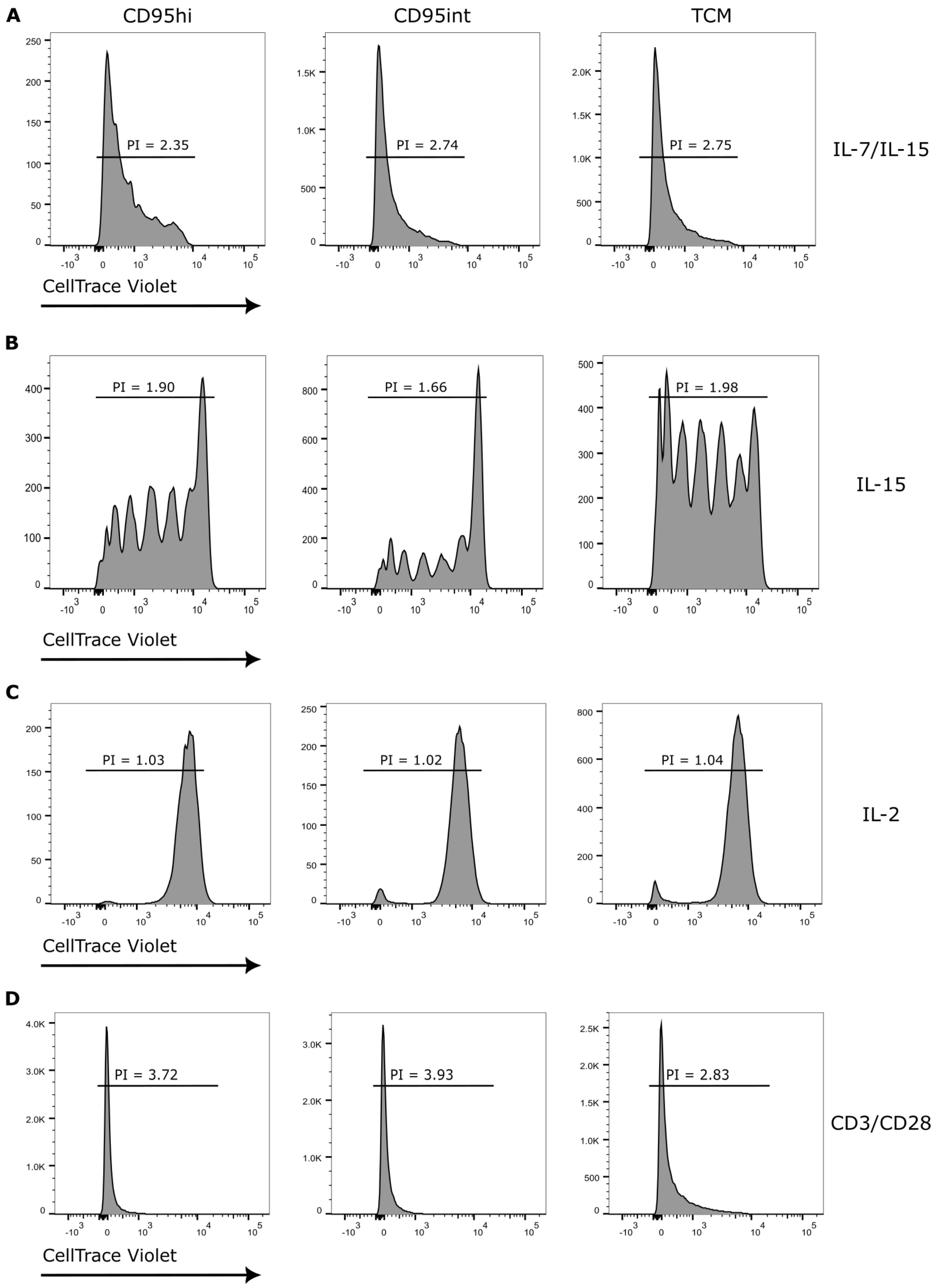

Figure D. CTV profile of sorted cells following 7 days *ex vivo* culture. Cell population ($CD8^+$ CD95hi T_{SCM}, CD95int T_{SCM}, T_{CM}) indicated at the top of each column, culture condition indicated on the right-hand side. Proliferation index (PI) is shown.

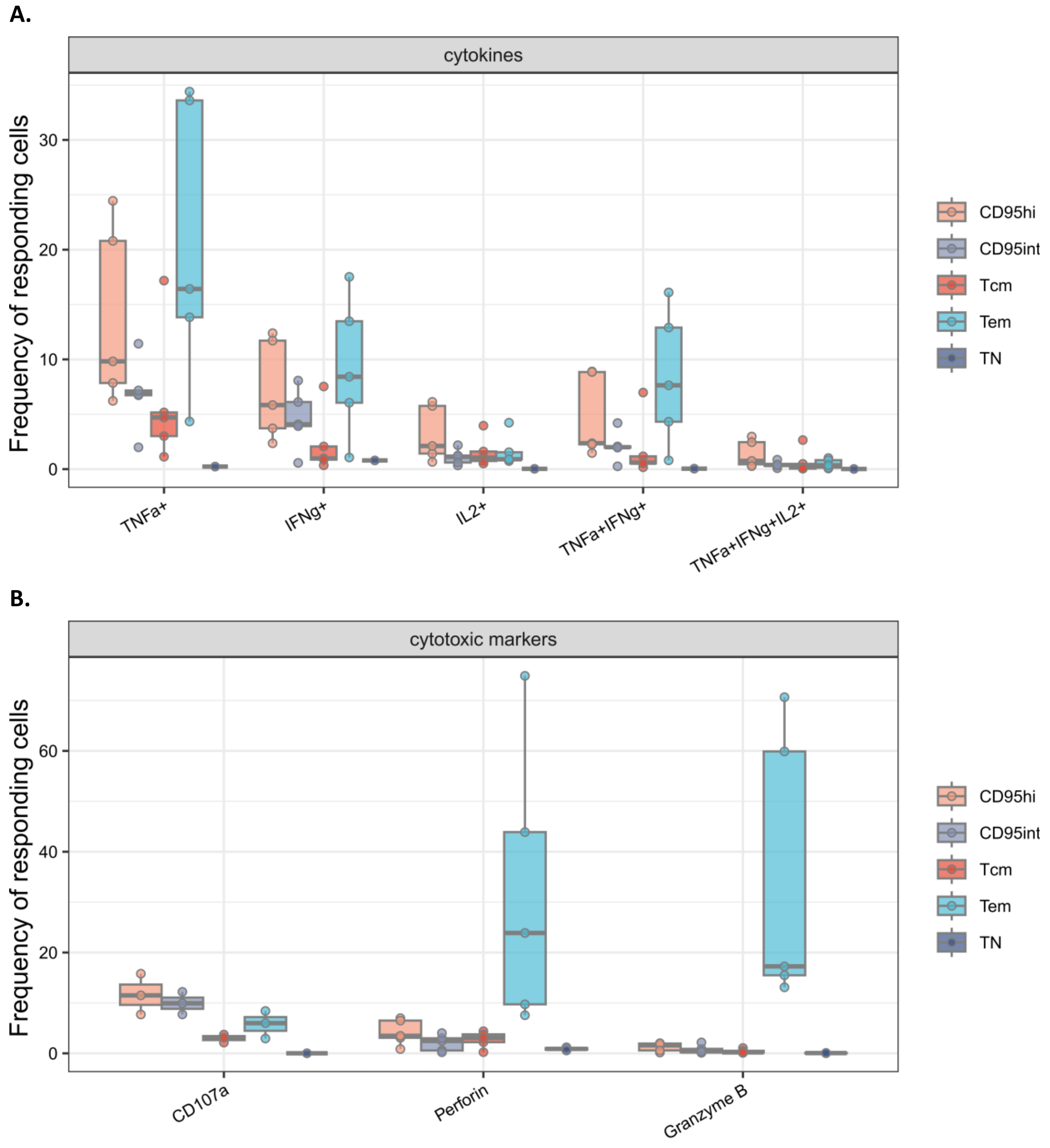

Figure E. CD95hi T_{SCM} cells exhibit increased functionality compared to both CD95int T_{SCM} cells and T_{CM} cells. Experimental conditions as for **Fig 2** but graph extended to show other subpopulations (T_{EM} and T_N) for comparison. **(A)** cytokine secretion **(B)** expression of markers associated with cytotoxicity.

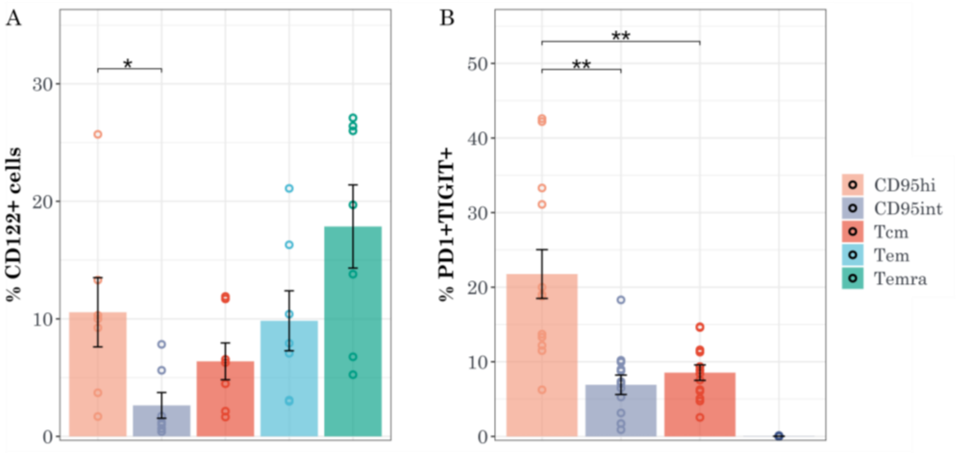

Figure F. Phenotypic characterisation of CD8^{+} memory subsets PBMCs from healthy donors from Cohort 1 were isolated and phenotyped by flow cytometry. Graphs summarise the percentage of **(A)** CD122^{+} and **(B)** PD1^{+}TIGIT^{+} cells amongst different subsets. Each dot represents one donor. Bars indicate mean ± sem. Stats: Wilcoxon * $P<0.05$ ** $P<0.01$. The data underlying this figure can be found in **S1 Data.**

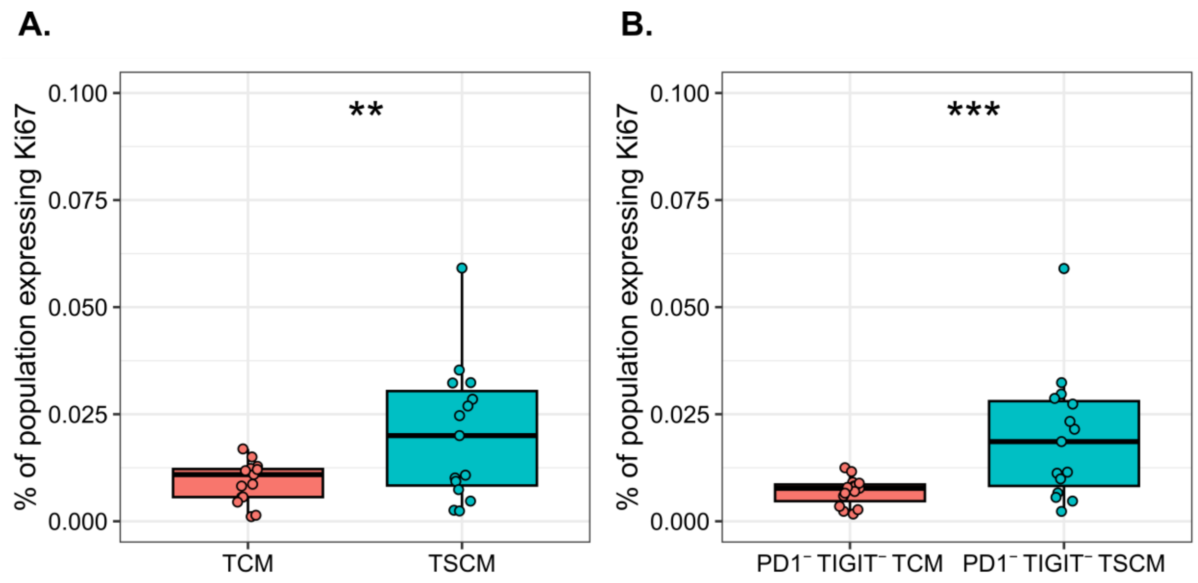

Figure G. Ki67 expression after exclusion of PD1+TIGIT+ cells. Ki67 expression was quantified in T_{CM} and T_{SCM} before (left) and after (right) exclusion of "exhausted" $PD1^{+}TIGIT^{+}$ cells. We found no evidence that exclusion of the exhausted $PD1^{+}TIGIT^{+}$ T_{SCM} and exhausted $PD1^{+}TIGIT^{+}$ T_{CM} cells made Ki67 expression in the remaining T_{SCM} and T_{CM} populations more similar. If anything, Ki67 expression in T_{SCM} and T_{CM} became more dissimilar, not less, upon exclusion of $PD1^{+}TIGIT^{+}$cells. The data underlying this figure can be found in **S1 Data.**

A.

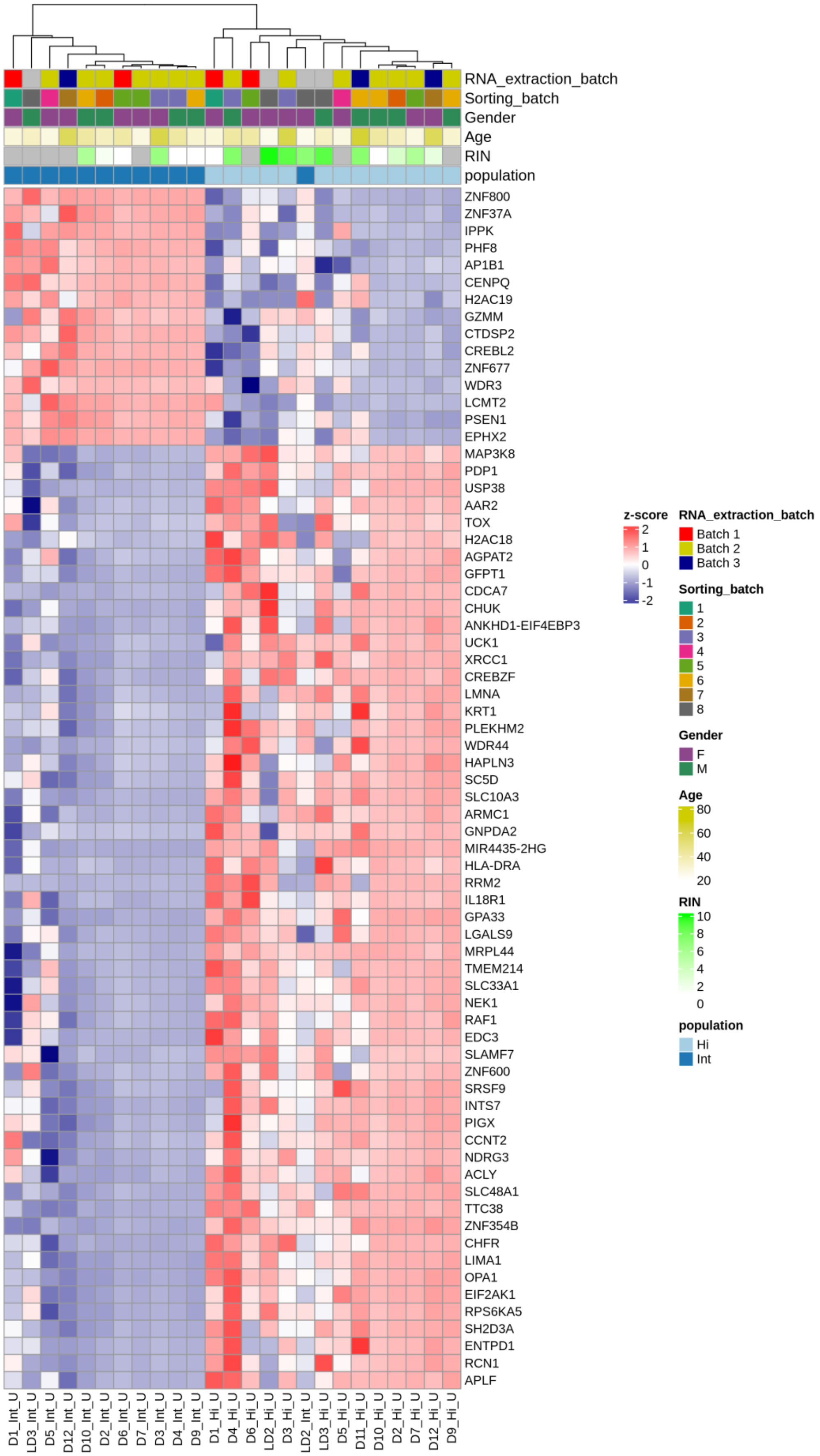
RNA_extraction_batch
Sorting_batch
Gender
Age
RIN
population
ZNF800
ZNF37A
IPPK
PHF8
AP1B1
CENPQ
H2AC19
GZMM
CTDSP2
CREBL2
ZNF677
WDR3
LCMT2
PSEN1
EPHX2
MAP3K8
PDP1
USP38
AAR2
TOX
H2AC18
AGPAT2
GFPT1
CDCA7
CHUK
ANKHD1-EIF4EBP3
UCK1
XRCC1
CREBZF
LMNA
KRT1
PLEKHM2
WDR44
HAPLN3
SC5D
SLC10A3
ARMC1
GNPDA2
MIR4435-2HG
HLA-DRA
RRM2
IL18R1
GPA33
LGALS9
MRPL44
TMEM214
SLC33A1
NEK1
RAF1
EDC3
SLAMF7
ZNF600
SRSF9
INTS7
PIGX
CCNT2
NDRG3
ACLY
SLC48A1
TTC38
ZNF354B
CHFR
LIMA1
OPA1
EIF2AK1
RPS6KA5
SH2D3A
ENTPD1
RCN1
APLF
D1_Int_U
LD3_Int_U
D5_Int_U
D12_Int_U
D10_Int_U
D2_Int_U
D6_Int_U
D7_Int_U
D3_Int_U
D4_Int_U
D9_Int_U
D1_Hi_U
D4_Hi_U
D6_Hi_U
LD2_Hi_U
D3_Hi_U
LD2_Int_U
LD3_Hi_U
D5_Hi_U
D11_Hi_U
D10_Hi_U
D2_Hi_U
D7_Hi_U
D12_Hi_U
D9_Hi_U
z-score
2
1
0
-1
-2
RNA_extraction_batch
Batch 1
Batch 2
Batch 3
Sorting_batch
1
2
3
4
5
6
7
8
Gender
F
M
Age
80
60
40
20
RIN
10
8
6
4
2
0
population
Hi
Int

B

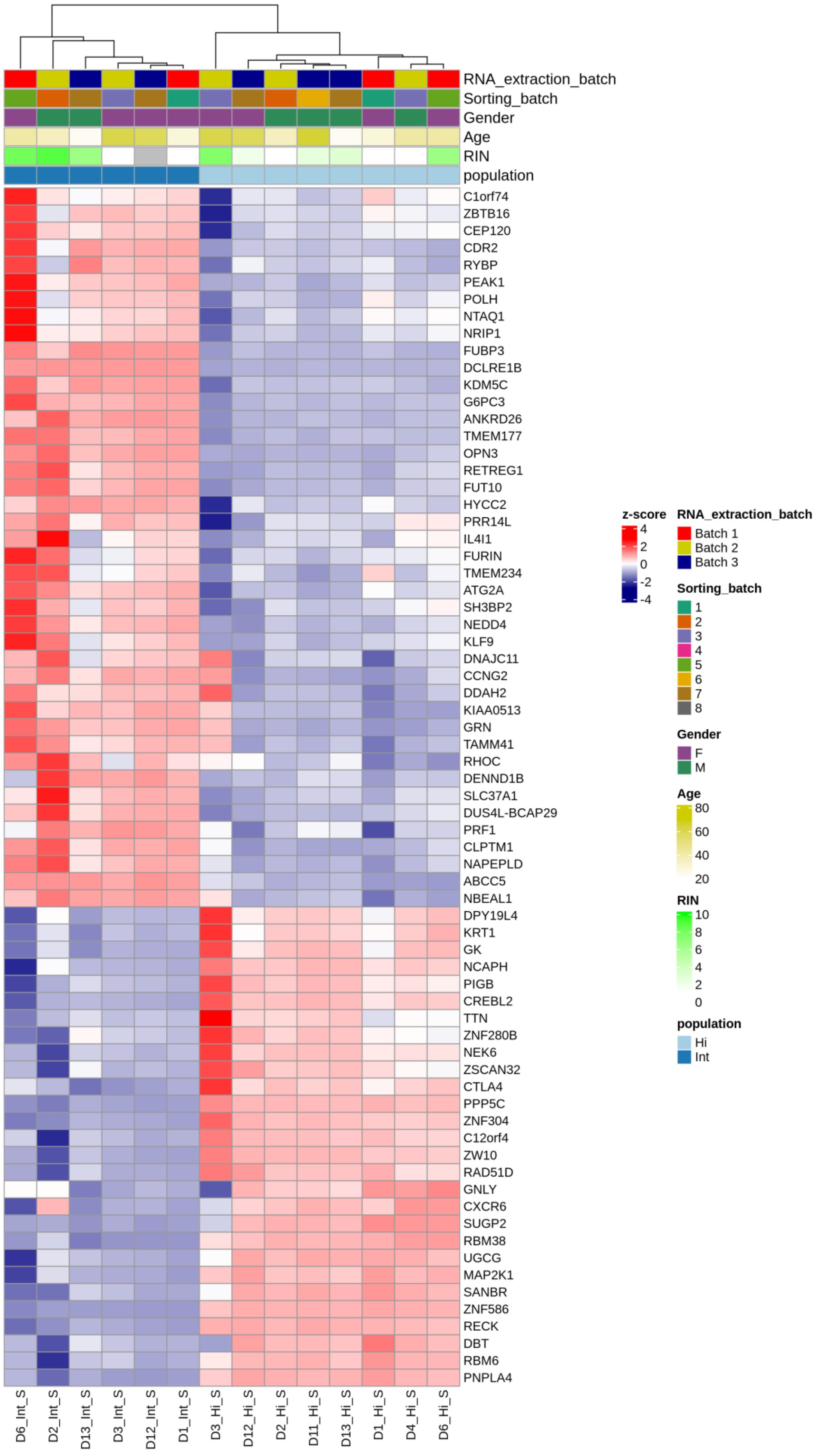

RNA_extraction_batch
Sorting_batch
Gender
Age
RIN
population
C1orf74
ZBTB16
CEP120
CDR2
RYBP
PEAK1
POLH
NTAQ1
NRIP1
FUBP3
DCLRE1B
KDM5C
G6PC3
ANKRD26
TMEM177
OPN3
RETREG1
FUT10
HYCC2
PRR14L
IL4I1
FURIN
TMEM234
ATG2A
SH3BP2
NEDD4
KLF9
DNAJC11
CCNG2
DDAH2
KIAA0513
GRN
TAMM41
RHOC
DENND1B
SLC37A1
DUS4L-BCAP29
PRF1
CLPTM1
NAPEPLD
ABCC5
NBEAL1
DPY19L4
KRT1
GK
NCAPH
PIGB
CREBL2
TTN
ZNF280B
NEK6
ZSCAN32
CTLA4
PPP5C
ZNF304
C12orf4
ZW10
RAD51D
GNLY
CXCR6
SUGP2
RBM38
UGCG
MAP2K1
SANBR
ZNF586
RECK
DBT
RBM6
PNPLA4
D6_Int_S
D2_Int_S
D13_Int_S
D3_Int_S
D12_Int_S
D1_Int_S
D3_Hi_S
D12_Hi_S
D2_Hi_S
D11_Hi_S
D13_Hi_S
D1_Hi_S
D4_Hi_S
D6_Hi_S
z-score
4
2
0
-2
-4
RNA_extraction_batch
Batch 1
Batch 2
Batch 3
Sorting_batch
1
2
3
4
5
6
7
8
Gender
F
M
Age
80
60
40
20
RIN
10
8
6
4
2
0
population
Hi
Int

Figure H. Heat maps of the 70 most differentially expressed genes.
A. Between unstimulated CD95int T_{SCM} and CD95hi T_{SCM} and **B.** between stimulated CD95int T_{SCM} and CD95hi T_{SCM}.

The data underlying this figure can be found in **S1 Data.**

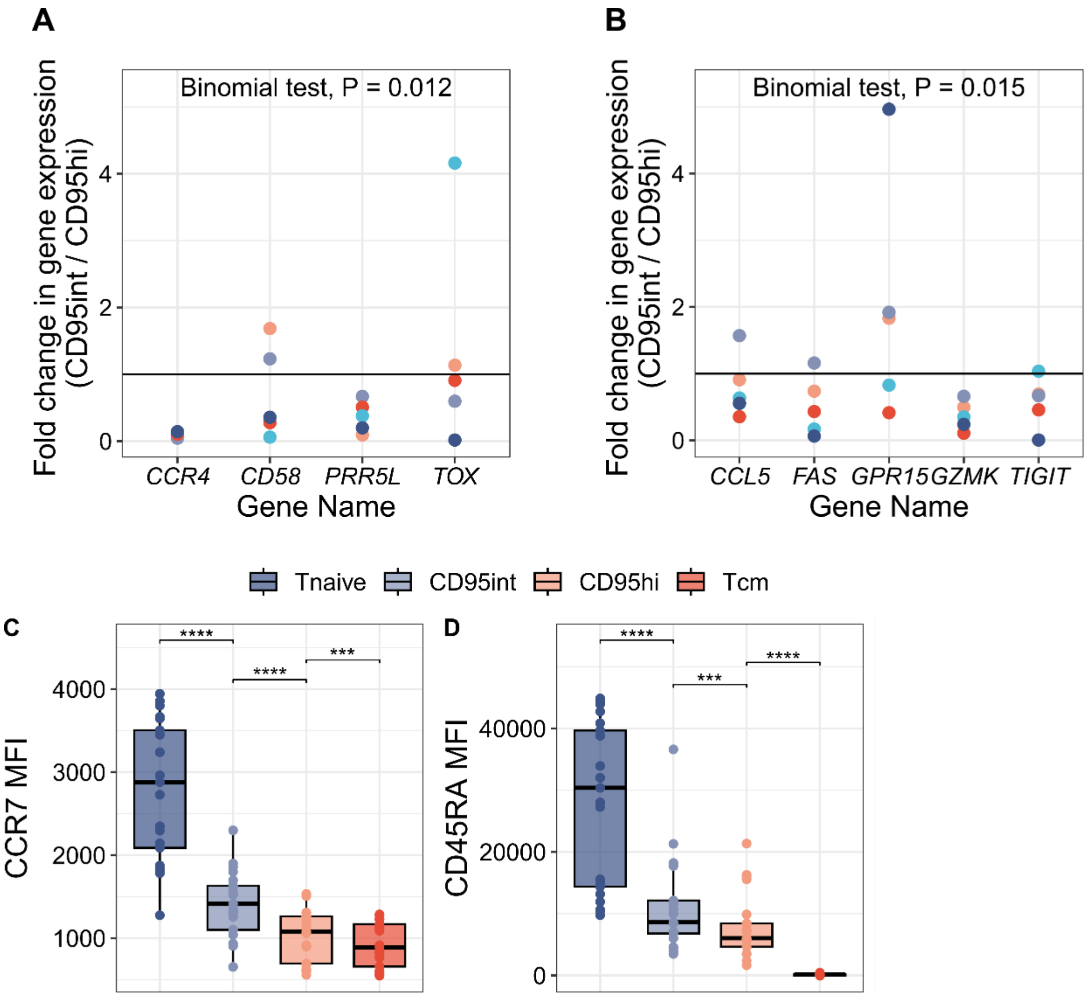

Figure I. CD95hi cells are transcriptionally and translationally closer to T_{CM} whilst CD95int more closely resemble T_N.

Gattinoni et al. identified genes enriched in T_{CM} cells compared to T_{SCM} cells and genes enriched in T_{SCM} cells compared to T_N cells. We selected, a priori, a small number of genes (9) from each list for analysis by qPCR. We focussed on CCR4, CD58, PRR5L and TOX as examples of genes overexpressed in T_{CM} compared to T_{SCM} cells and CCL5, FAS, GPR15, GZMK and TIGIT as examples of genes overexpressed in T_{SCM} compared to T_N cells. **(A, B)** We compared expression of these two gene sets between sorted CD95int and CD95hi T_{SCM} cells. The y axis shows the fold change in the expression of each gene in the CD95int subset compared to the CD95hi subset calculated per gene and per donor, one dot per donor (N=5). Values above the horizontal line (at y=1) indicated higher expression in CD95int cells than CD95hi cells; values below the line indicate higher expression in CD95hi cells. It can be seen than genes that tend to be overexpressed in T_{CM} compared to T_{SCM} (panel A), are overexpressed in CD95hi cells compared to CD95int cells (i.e. CD95hi gene expression more closely resembles that of TCM than it does for CD95int cells). Similarly, genes that tend to be overexpressed in T_{SCM}

compared to T_N (panel B) are also overexpressed in in CD95hi cells compared to CD95int cells (i.e. CD95int gene expression more closely resemble that of T_N than CD95int cells). Two tailed binomial test to test the null hypothesis that CD95int and CD95hi are equidistant from T_{CM} and that CD95int and CD95hi are equidistant from T_N was conducted. The null was rejected in both cases (P=0.012 and P=0.015 respectively). Median Fluorescence intensity for (C) CCR7 and (D) CD45RA was measured for CD95hi, CD95int, TCM and TN subsets from 21 healthy individuals. In C & D, bars represent the median, the box the interquartile range (IQR) and whiskers show lower quartile -1.5*IQR and upper quartile +1.5*IQR; all data points (including outliers) are shown by filled circles. Statistics: Paired Wilcoxon. CCR7: T_N v CD95int P=1x10-6, CD95int v CD95hi P=1x10-6, CD95hi v T_{CM} P=0.0006. CD45RA: T_N v CD95int P=1x10-6, CD95int v CD95hi P=0.0006, CD95hi v T_{CM} P=1x10-6. *** P<0.001, **** P<0.0001. Number of independent tests ≤6. All subjects from Cohort 1.

The data underlying this figure can be found in **S1 Data.**

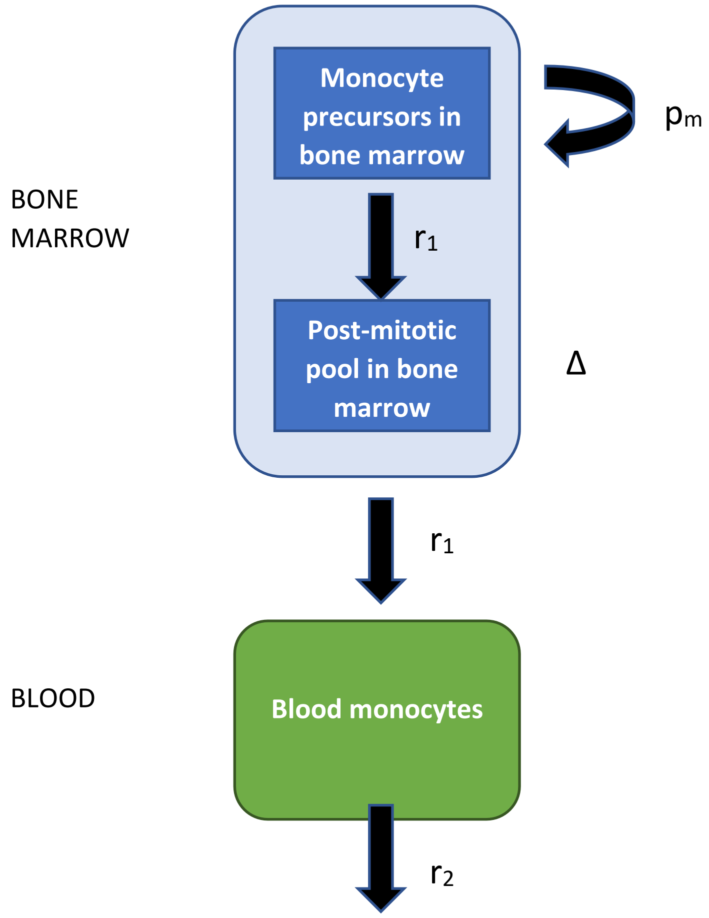

Figure J. Schematic of Mechanistic Model to Describe Monocyte Kinetics. Here p_m is the proliferation rate of precursors, r_1 is the rate of exit from the mitotic pool in bone marrow, Δ is the time spent in the post-mitotic pool in bone marrow and r_2 is the rate of disappearance of blood monocytes (death and long-term exit from the blood).

A.

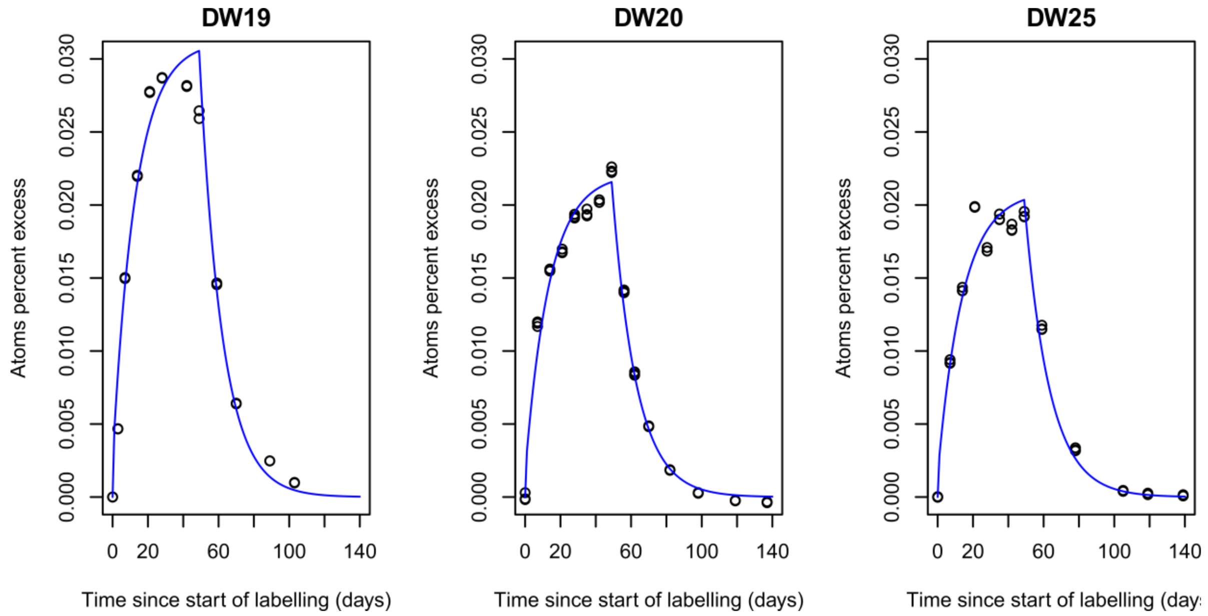

B.

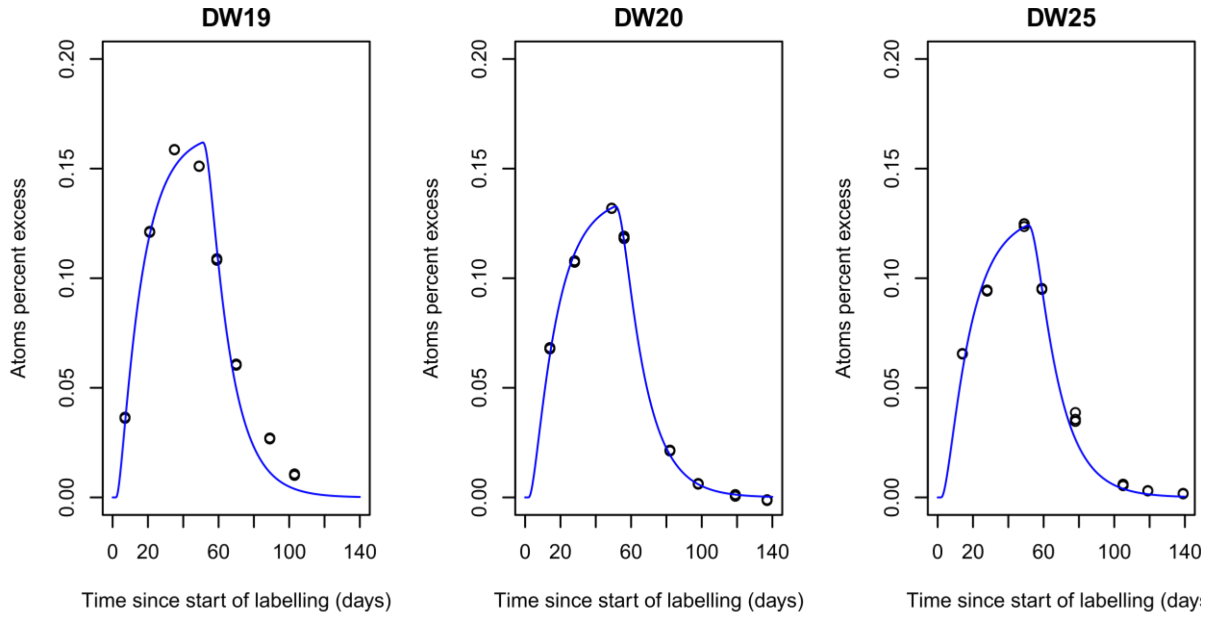

Figure K. Best fits of the models to the saliva and monocyte data. (A). Best fit of the model to the saliva data from the three labelled individuals. **(B).** Best fit of the model to the saliva data from the three labelled individuals. In all cases black dots represent the observed data, blue lines the best fit predictions.

The data underlying this figure can be found in **S1 Data.**

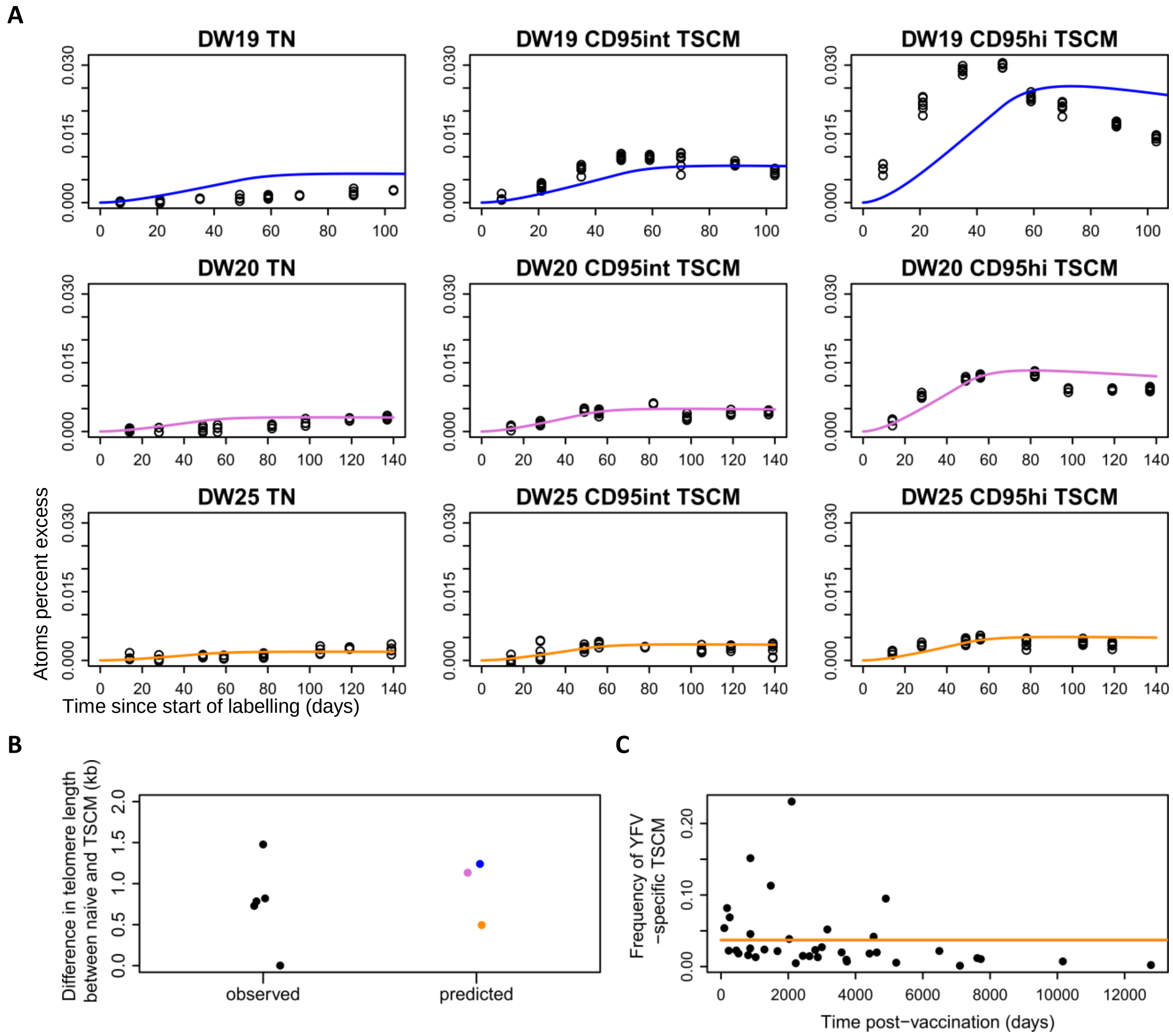

Figure L. Fit of Model A (independent homogeneous populations) to all data (labelling, telomere, YFV) simultaneously. (A) Fit of model to the labelling data (each individual in a different row, each cell population in a different column); dots represent data, lines represent model predictions. **(B)** Fit of model to the telomere data. Observed differences in mean telomere length between T_N and T_{SCM} for 5 individuals shown on the left in black, model predictions for each individual shown by coloured points on the right (DW19 in blue, DW20 in pink, DW25 in orange). **(C)** Fit of the model to the YFV data. Observed data shown by black points, model predictions for each individual shown by coloured lines (DW19 in blue, DW20 in pink, DW25 in orange); in this case the three predictions overlay and so only one line can be seen. The data underlying this figure can be found in **S1 Data.**

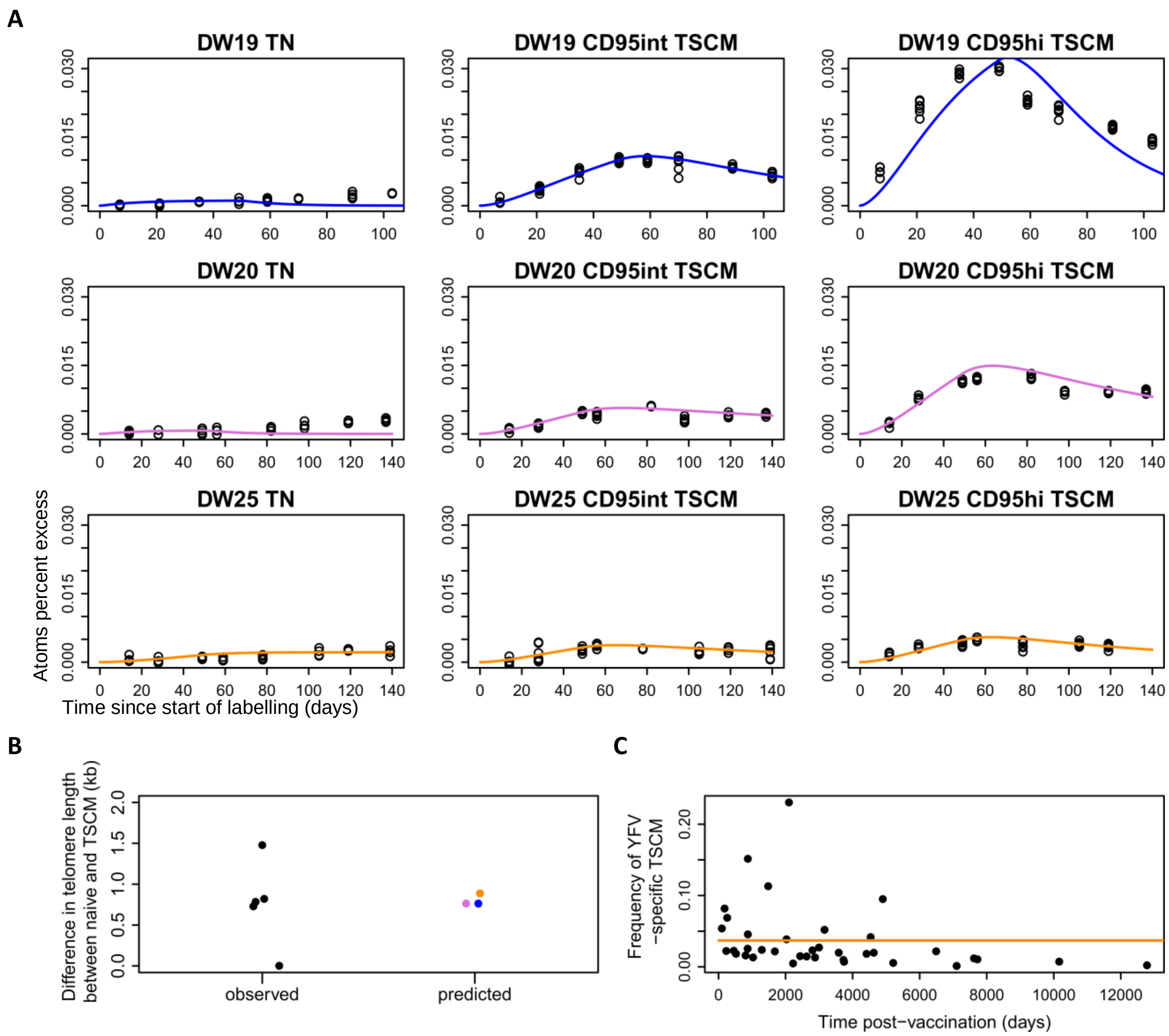

Figure M. Fit of Model B (independent heterogeneous populations) to all data (labelling, telomere, YFV) simultaneously. (A) Fit of model to the labelling data (each individual in a different row, each cell population in a different column); dots represent data, lines represent model predictions. **(B)** Fit of model to the telomere data. Observed differences in mean telomere length between T_N and T_{SCM} for 5 individuals shown on the left in black, model predictions for each individual shown by coloured points on the right (DW19 in blue, DW20 in pink, DW25 in orange). **(C)** Fit of the model to the YFV data. Observed data shown by black points, model predictions for each individual shown by coloured lines (DW19 in blue, DW20 in pink, DW25 in orange); in this case the three predictions overlay and so only one line can be seen. The data underlying this figure can be found in **S1 Data.**

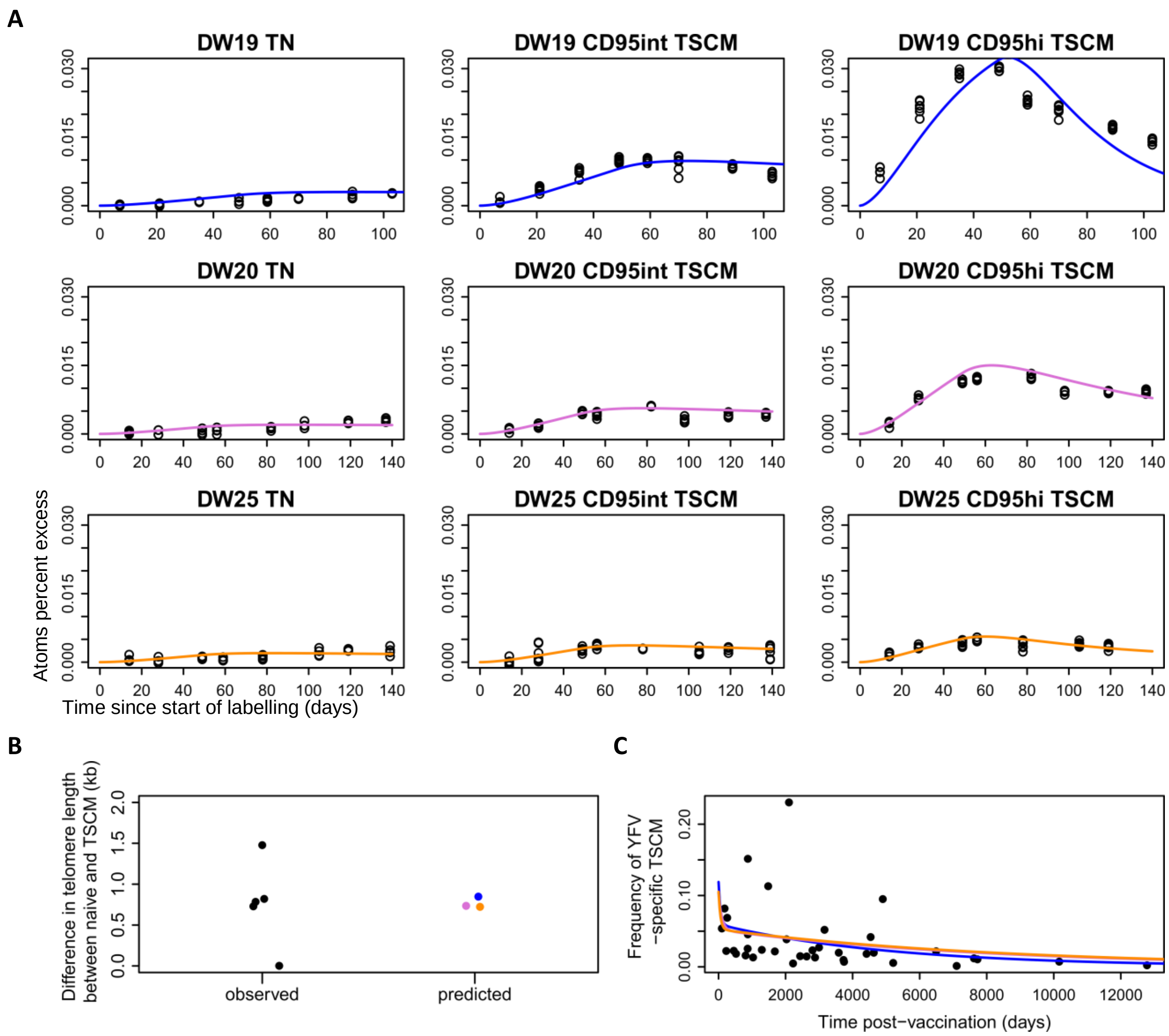

Figure N. Fit of Model C (fork) to all data (labelling, telomere, YFV) simultaneously. (A) Fit of model to the labelling data (each individual in a different row, each cell population in a different column); dots represent data, lines represent model predictions. **(B)** Fit of model to the telomere data. Observed differences in mean telomere length between T_N and T_{SCM} for 5 individuals shown on the left in black, model predictions for each individual shown by coloured points on the right (DW19 in blue, DW20 in pink, DW25 in orange). **(C)** Fit of the model to the YFV data. Observed data shown by black points, model predictions for each individual shown by coloured lines (DW19 in blue, DW20 in pink, DW25 in orange); in this case the predictions from DW20 and DW25 overlay. The data underlying this figure can be found in **S1 Data.**

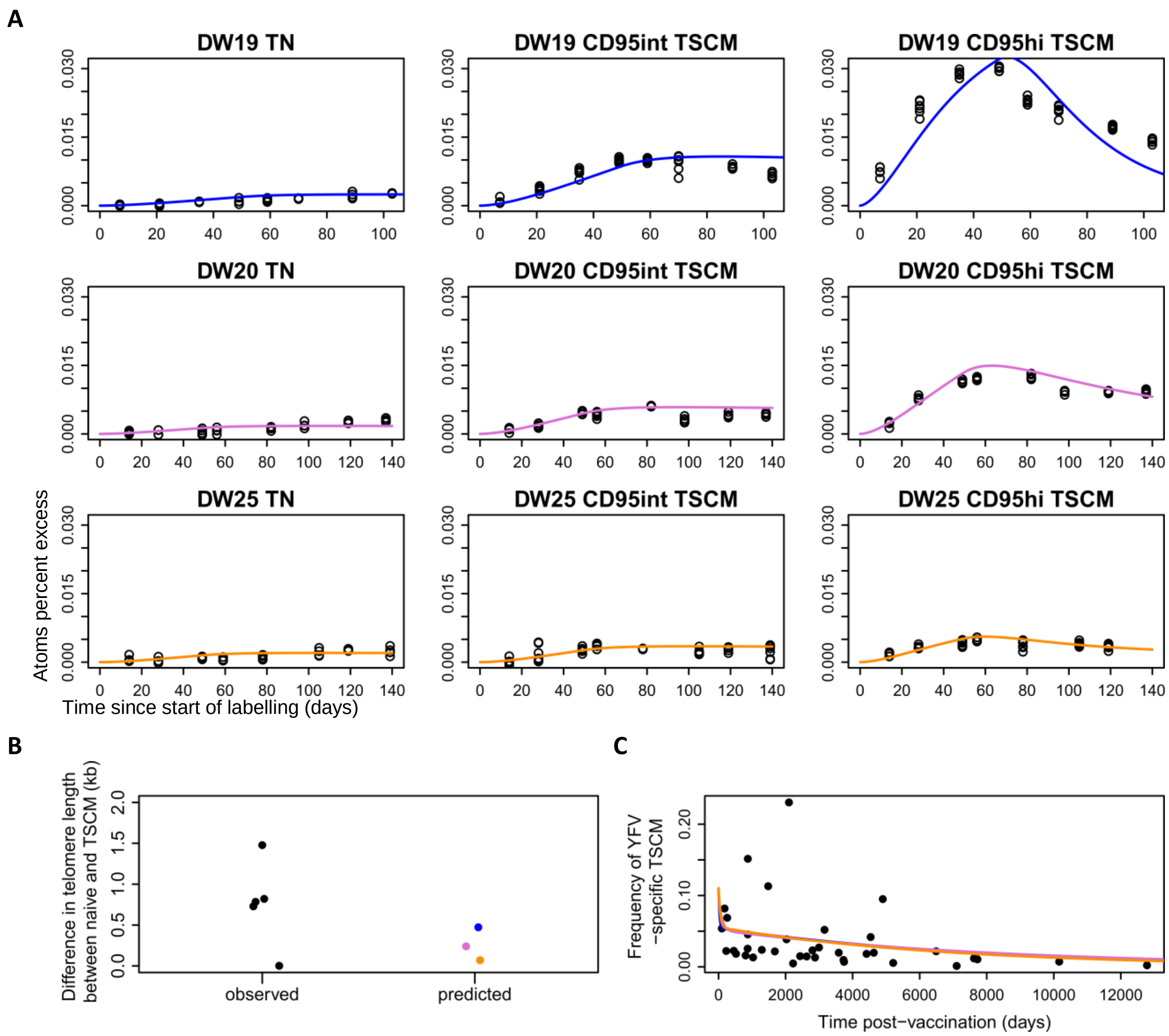

Figure O. Fit of Model E (linear, CD95hi first) to all data (labelling, telomere, YFV) simultaneously. (A) Fit of model to the labelling data (each individual in a different row, each cell population in a different column); dots represent data, lines represent model predictions. **(B)** Fit of model to the telomere data. Observed differences in mean telomere length between T_N and T_{SCM} for 5 individuals shown on the left in black, model predictions for each individual shown by coloured points on the right (DW19 in blue, DW20 in pink, DW25 in orange). **(C)** Fit of the model to the YFV data. Observed data shown by black points, model predictions for each individual shown by coloured lines (DW19 in blue, DW20 in pink, DW25 in orange); in this case the predictions overlay and only one line can be seen. The data underlying this figure can be found in **S1 Data.**

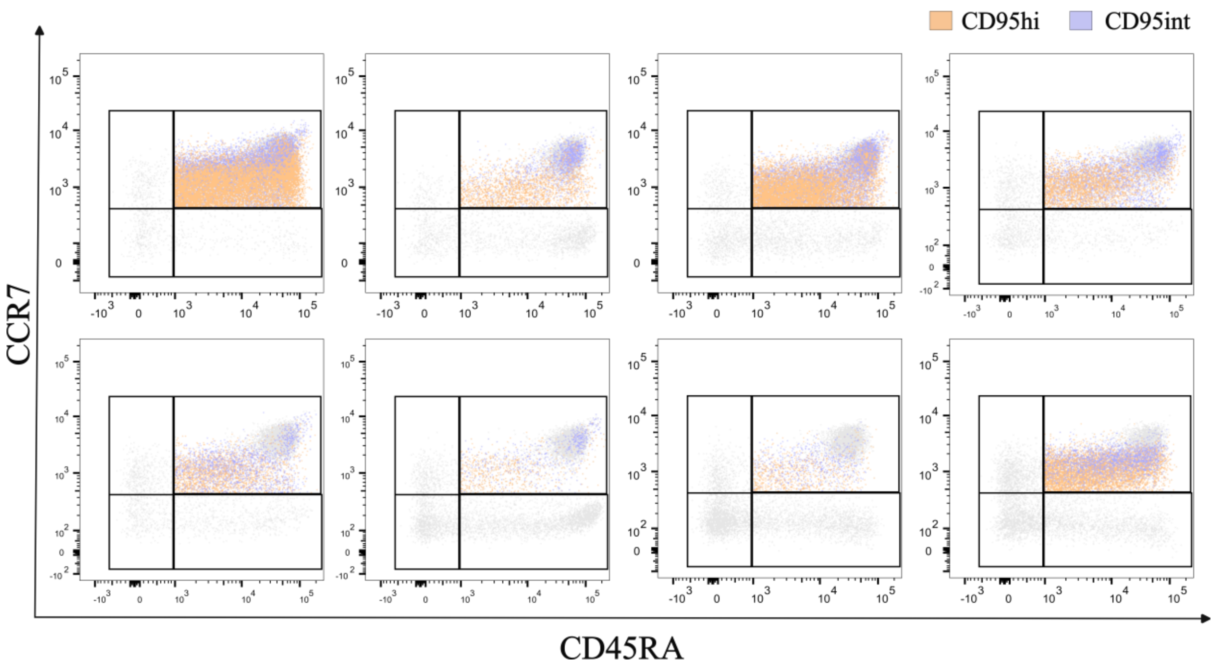

Figure P. CD45A and CCR7 expression of the CD95hi T_{SCM} and CD95int T_{SCM} population. CD95int T_{SCM} (blue) and CD95hi T_{SCM} (orange) subpopulations were backgated onto the CD45RA/CCR7 axis. It can be seen that CD95int T_{SCM} tend to have higher levels of CD45RA and CCR7 expression than CD95hi T_{SCM}. See also **Figure I (Panels C & D) in S1 Text**.

The data underlying this figure can be found in **S1 Data.**

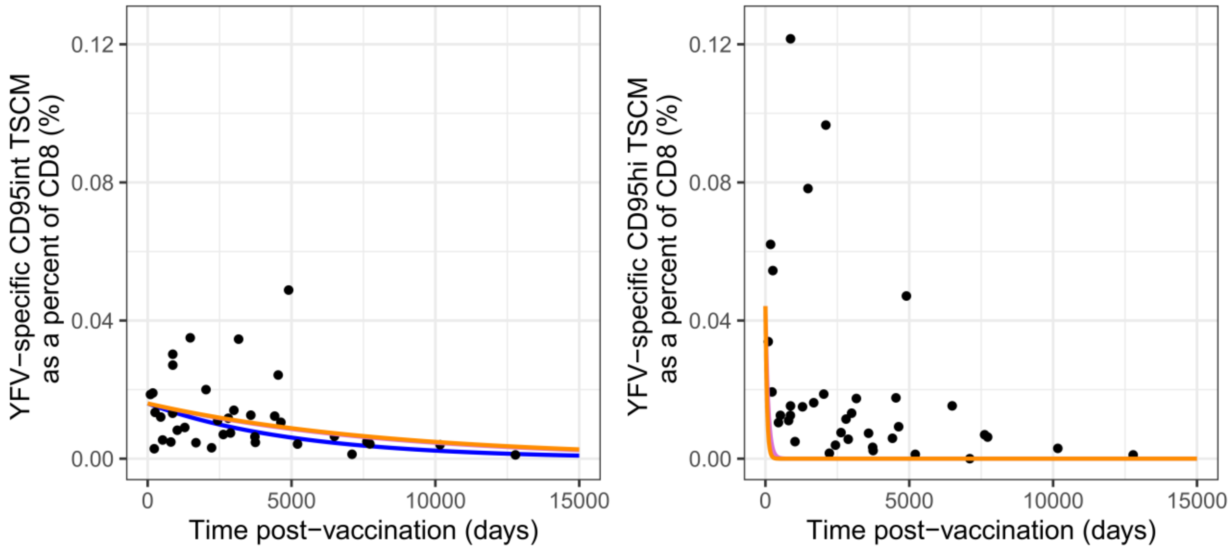

Figure Q. Predictions of Model C (fork) overlaid (NOT fitted) onto a novel data set. The fork model and best fit parameters were used to predict the long-term dynamics of YFV-specific CD95int T_{SCM} (left) and CD95hi T_{SCM} (right) for each individual (shown by coloured lines: DW19 in blue, DW20 in pink, DW25 in orange). For CD95int the predictions for DW20 and DW25 overlay so only two lines can be seen, for CD95hi all three predictions overlay and so only one line can be seen. The predictions are plotted against but not fitted to a proxy for CD95int and CD95hi T_{SCM} frequencies measured in the cross-sectional cohort post-vaccination (the same cohort for which total T_{SCM} were fitted). It can be seen that the predictions for the CD95hi population fail, underestimating virtually all the data points.

The data underlying this figure can be found in **S1 Data.**

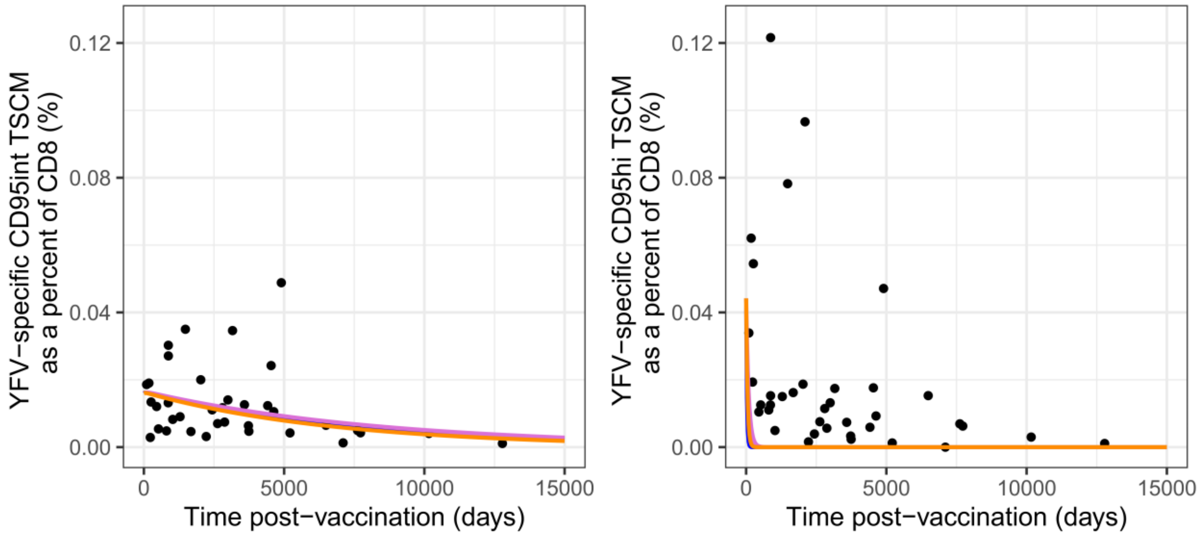

Figure R. Predictions of Model E (linear CD95hi first) overlaid (NOT fitted) onto a novel data set. The linear (CD95hi first) model and best fit parameters were used to predict the long-term dynamics of YFV-specific CD95int T_{SCM} (left) and CD95hi T_{SCM} (right) for each individual (shown by coloured lines: DW19 in blue, DW20 in pink, DW25 in orange). For both CD95int and CD95hi the predictions for all three individuals overlay and so only one line can be seen. The predictions are plotted against but not fitted to a proxy for CD95int and CD95hi T_{SCM} frequencies measured in the cross-sectional cohort post-vaccination (the same cohort for which total T_{SCM} were fitted). It can be seen that the predictions for the CD95hi population fail, underestimating virtually all the data points.

The data underlying this figure can be found in **S1 Data.**

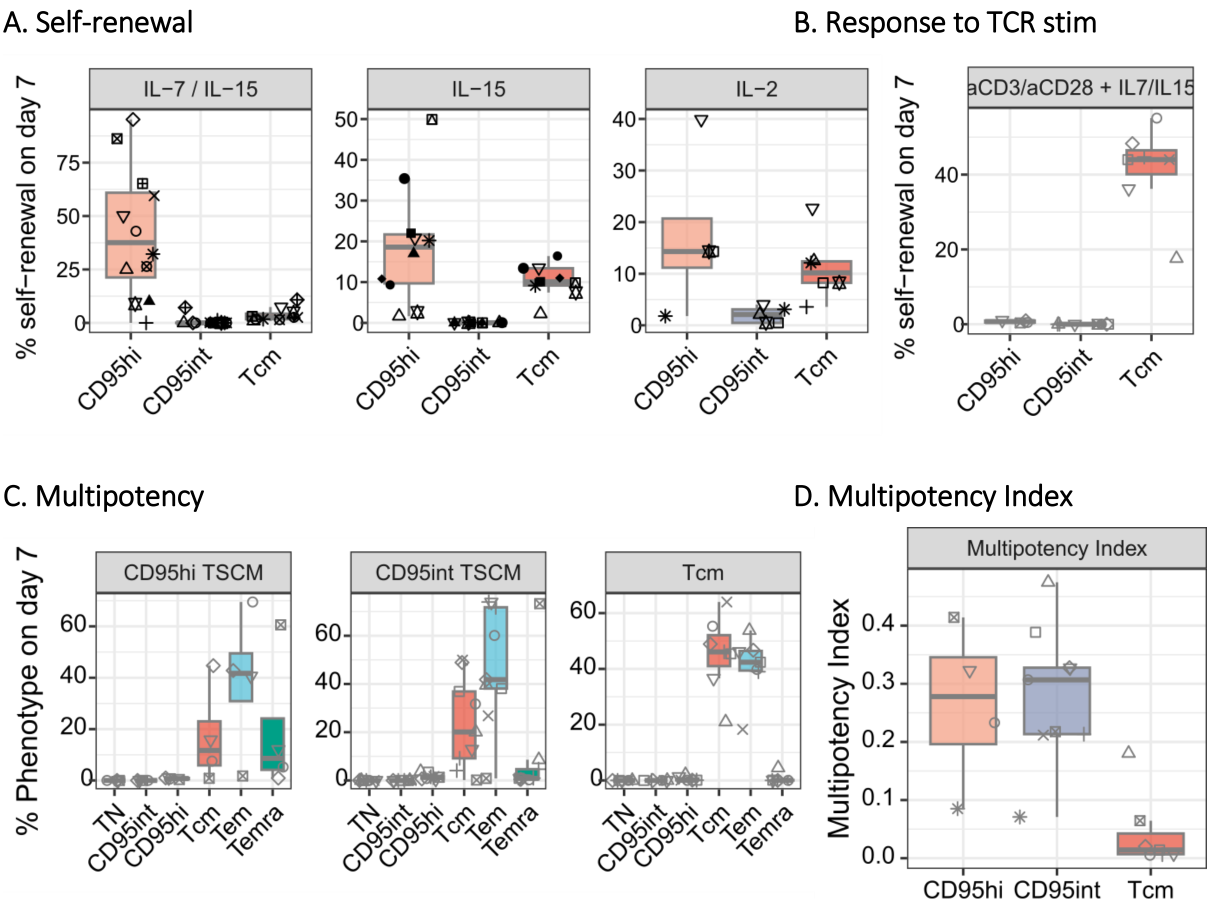

Figure S. Duplicate of Fig 2 (main text) showing unique identifier for each donor in response to request from reviewer. The data underlying this figure can be found in **S1 Data**.

1. Geginat J, Lanzavecchia A, Sallusto F. Proliferation and differentiation potential of human CD8+ memory T-cell subsets in response to antigen or homeostatic cytokines. Blood. 2003;101(11):4260-6. doi: 10.1182/blood-2002-11-3577.
2. Bell EB, Sparshott SM. Interconversion of CD45R subsets of CD4 T cells in vivo. Nature. 1990;348(6297):163-6. doi: 10.1038/348163a0. PubMed PMID: 1978253.
