# Supplementary material for: Two distinct subpopulations of human stem-like memory T cells exhibit complementary roles in self-renewal and clonal longevity
Source: PLoS Biol. 2025 Jun 20;23(6):e3003179. doi: 10.1371/journal.pbio.3003179 (PMC12251145; doi:10.1371/journal.pbio.3003179)
Supplement: S2 Text — Table A. Antibodies used for flow cytometry and sorting. Table B. Human TaqMan probes used for RT-PCR. Table C. RNAseq Experimental Design. Table D. Saliva and monocyte parameters. Table E. Parameters in the models for fitting labelling, telomere and YF-vaccination data. Table F. Parameters obtained by fitting model A (indep homog) to labelling, telomere and YFV data. Table G. Parameters obtained by fitting model B (indep heterog) to labelling, telomere and YFV data. Table H. Parameters obtained by fitting model C (fork) to labelling, telomere and YFV data. Table I. Parameters obtained by fitting model E (linear, CD95hi first) to labelling, telomere and YFV data. Table J. Summary of the fits of the 5 models to the labelling, telomere and YFV vaccination data. Table K. Summary of the fits of the 5 models to the labelling data only. Table L. Summary of the comparison of the 5 model predictions with the unseen, unfitted YFV vaccination data. (PDF) [file pbio.3003179.s002.pdf]

S2 Text
(Supplementary Tables)

Antibody	Fluorochrome	Clone	Dilution	Supplier
Anti-human Ki67	BV421	Ki67	1:200	BD Biosciences
Anti-human Perforin	BV421	dG9	1:50	BioLegend
CellTrace™ Violet	BV421	-	50uM	Thermo Fisher
Anti-human IFNg	V500	B27	1:300	BD Biosciences
Anti-human CD19	V500	HIB19	1:200	BD Biosciences
Anti-human CD14	V500	M5E2	1:100	BD Biosciences
Anti-human CD27	QDot605	CLB-27/1	1:200	Thermo Fisher
Anti-human CD122	BV650	Mik-b3	1:50	BD Biosciences
Anti-human CD8	BV711	RPA-T8	1:100	BioLegend
Anti-human CD3	BV785	SK7	1:100	BioLegend
Anti-human CCR7	FITC	150503	1:25	BD Biosciences
Anti-human CD95	PE/Cy5	DX2	1:50	BioLegend
Anti-human TIGIT	PerCP-e710	MBAS43	1:25	BD Biosciences
Anti-human Granzyme B	PE	PE	1:300	BD Biosciences
Anti-human TNFa	PE	MAb11	1:300	BioLegend
Anti-human CD45RA	ECD	2H4	1:25	Beckman Coulter
Anti-human CD4	PE-Cy5.5	MHCD0418	1:100	Thermo Fisher
Anti-human PD-1	PE/Cy7	EH12.H7	1:150	BioLegend
Anti-human CD107a	PE/Cy7	H4A3	1:200	BioLegend
Anti-human IL-2	PE/Cy7	MQ1-17H12	1:100	BD Biosciences
Live/Dead	Near-Infrared	-	1:1000	Thermo Fisher

Table A. Antibodies used for flow cytometry and sorting.

Gene	TaqMan Assay ID
18S rRNA	Hs99999901_s1
BACH2	Hs00935338_m1
BCL6	Hs00153368_m1
CCL5	Hs00982282_m1
CCR4	Hs00747615_s1
CD58	Hs00156385_m1
CXCR5	Hs00173527_m1
ENTPD1	Hs00969556_m1
EOMES	Hs00172872_m1
FAS	Hs00236330_m1
FOXO1	Hs00231106_m1
GPR15	Hs00922903_s1
GZMK	Hs00157878_m1
HAVCR2	Hs00958618_m1
ID3	Hs00171409_m1
ID2	Hs04187239_m1
KLF2	Hs07291763 gH
KLRG1	Hs00195153_m1
KLRK1	Hs00183683_m1
LEF1	Hs01547250_m1
MYB	Hs00920556_m1
PDCD1	Hs00169472_m1
PRR5L	Hs01029928_m1
SELL	Hs00174151_m1
TBx21	Hs00894392_m1
TCF7	Hs00175273_m1
TIGIT	Hs00545087_m1
TIMD4	Hs00293316_m1
TOX	Hs01055573_m1
XCL1	Hs00751481_s1

Table B. Human TaqMan probes used for RT-PCR.

	ID	Gender	Age	Sorting batch	RNA extraction batch	STIM						UNSTIM					
						Hi	Int	Tcm	Tem	Temra	Tn	Hi	Int	Tcm	Tem	Temra	Tn
1	LD2	F	25	8	NA												
2	D5	F	27	4	Batch 2												
3	D7	F	27	5	Batch 2												
4	D1	F	29	1	Batch 1												
5	D6	F	41	5	Batch 1												
6	D12	F	58	7	Batch 3												
7	D3	F	61	3	Batch 2												
8	D13	M	23	7	Batch 3												
9	D9	M	31	6	Batch 2												
10	LD3	M	34	8	NA												
11	D2	M	35	2	Batch 2												
12	D10	M	42	6	Batch 2												
13	D4	M	42	3	Batch 2												
14	D11	M	65	6	Batch 3												
**Total**						8	8	4	5	5	5	14	14	4	5	5	5

Table C. RNAseq Experimental Design. Blood samples from 14 different donors were processed sorted in different batches for T_N, CD95hi T_{SCM}, CD95int T_{SCM}, T_{CM}, T_{EM} and T_{EMRA}. RNA was extracted for a subset of samples (highlighted in gray) before or after stimulation (see methods) for RNA sequencing.

ID	f	δ (per day)	r_2 (per day)	b_w
DW19	0.062	0.077	0.38	5.34
DW20	0.044	0.073	0.31	6.22
DW25	0.042	0.071	0.29	6.18

Table D. Saliva and monocyte parameters. For each of the three individuals the best estimates of the model parameters obtained by fitting the models to the stable isotope labelling data from saliva and monocytes is shown. r_2 is very poorly identified and only included for completeness, these estimates are unreliable.

Model ID	Model	Eliminated parameters	Free Parameters	Number of free parameters
A	Indep homog.	d_N, d_1, d_2	p_N, p_1, p_2, A	4
B	Indep heterog.	None	p_N, p_1, p_2, d_N*, d_1*, d_2*, A	7
C	fork	d_N, d_1, d_2	p_N, p_1, p_2, k, Δ_N, f, A	7
D	linear TN-> Tint->Thi	d_N, d_1, d_2	p_N, p_1, p_2, Δ_N, Δ_1, k, A	7
E	linear TN->Thi->Tint	d_N, d_1, d_2	p_N, p_1, p_2, Δ_N, Δ_1, k, A	7

Table E. Parameters in the models for fitting labelling, telomere and YF-vaccination data. For each of the 5 models considered, the parameters that were eliminated by equilibrium constraints, the free parameters that were fitted and the number of free parameters is shown. For models C, D and E the ratio of the size of the CD95int population to the T_N population and the ratio of the size of the CD95hi population to the T_N population were derived from the experimental data. Sketches of the five models are shown in **Fig 7**, the corresponding equations are in the methods. When fitting only the labelling data then the parameter *A* is not required, and the number of free parameters is reduced by 1 for each model; for Model C (fork) a reparameterization in the labelling only case further reduces the number of free parameters by 1 (to 5).

ID	pN (days^{-1})	SE pN (days^{-1})	p1 (days^{-1})	SE p1 (days^{-1})	p2 (days^{-1})	SE p2 (days^{-1})	A (% of CD8)	SE A (% of CD8)
DW19	8.0E-04	2.00E-04	1.0E-03	2.1E-04	3.6E-03	8.3E-05	0.018	0.002
DW20	4.7E-04	1.16E-04	7.6E-04	1.3E-04	2.2E-03	1.4E-04	0.018	0.002
DW25	3.0E-04	7.14E-05	5.6E-04	7.1E-05	8.4E-04	1.6E-05	0.019	0.002

Table F. Parameters obtained by fitting model A (indep homog) to labelling, telomere and YFV data. Table shows best fit estimates of the parameters and the corresponding standard errors (SE) obtained by the asymptotic covariance matrix method.

id	pN (days^{-1})	SE pN (days^{-1})	p1 (days^{-1})	SE p1 (days^{-1})	p2 (days^{-1})	SE p2 (days^{-1})	d*N (days^{-1})	SE d*N (days^{-1})	d*1 (days^{-1})	SE d*1 (days^{-1})	d*2 (days^{-1})	SE d*2 (days^{-1})	A (% of CD8)	SE A (% of CD8)
DW19	4.0E-03	6.7E-04	2.2E-03	6.7E-04	1.0E-02	1.3E-03	6.0E-01	5.6E-03	1.6E-02	9.5E-03	4.1E-02	6.4E-03	0.018	0.002
DW20	1.4E-03	5.7E-04	1.1E-03	4.0E-04	3.2E-03	8.7E-04	2.5E-01	2.3E-03	6.0E-03	5.6E-03	9.8E-03	2.4E-03	0.018	0.002
DW25	3.3E-04	1.3E-04	8.1E-04	1.6E-04	1.3E-03	1.8E-04	0.0E+00	6.2E-03	8.5E-03	3.9E-03	1.1E-02	3.6E-03	0.018	0.002

Table G. Parameters obtained by fitting model B (indep heterog) to labelling, telomere and YFV data. Table shows best fit estimates of the parameters and the corresponding standard errors (SE) obtained by the asymptotic covariance matrix method

id	pN (days^{-1})	SE pN (days^{-1})	p1 (days^{-1})	SE p1 (days^{-1})	p2 (days^{-1})	SE p2 (days^{-1})	ΔN (days^{-1})	SE ΔN (days^{-1})	f	SE f	k	SE k	A (% of CD8)	SE A (% of CD8)	Clonal half-life CD95int (days)	Clonal half-life CD95hi (days)
DW19	9.4E-04	7.1E-04	3.4E-03	7.1E-04	2.7E-02	6.7E-03	2.0E-04	1.6E-05	2.5E-02	5.2E-03	0.0	0.7	0.06	0.009	3779	39
DW20	9.1E-04	4.6E-04	2.7E-03	6.4E-04	1.4E-09	5.4E-03	3.1E-05	2.0E-05	1.5E-02	3.6E-03	3.3	1.6	0.05	0.009	6058	66
DW25	2.2E-03	8.9E-04	4.4E-03	1.5E-03	4.7E-09	4.6E-03	6.1E-04	2.4E-04	6.6E-03	1.7E-03	1.0	0.0	0.05	0.008	6166	37

Table H. Parameters obtained by fitting model C (fork) to labelling, telomere and YFV data. Table shows best fit estimates of the parameters and the corresponding standard errors (SE) obtained by the asymptotic covariance matrix method.

id	pN (days^{-1})	SE pN (days^{-1})	p2 (days^{-1})	SE p2 (days^{-1})	p1 (days^{-1})	SE p1 (days^{-1})	ΔN (days^{-1})	SE ΔN (days^{-1})	Δ1 (days^{-1})	SE Δ1 (days^{-1})	k	SE k	A (% of CD8)	SE A (% of CD8)	Clonal half-life CD95int (days)	Clonal half-life CD95hi (days)
DW19	3.0E-04	2.0E-04	1.1E-02	1.3E-01	1.4E-03	1.8E-04	4.0E-04	7.5E-05	3.1E-04	1.2E-04	0.00	5.16	0.05	0.008	5024	18
DW20	2.6E-04	1.2E-04	1.7E-08	1.3E-02	8.9E-04	1.3E-04	2.2E-04	6.6E-05	1.5E-04	8.0E-05	0.49	1.84	0.05	0.010	5454	64
DW25	3.2E-04	9.7E-05	1.5E-03	5.1E-02	5.6E-04	1.3E-04	1.4E-03	5.7E-04	1.5E-04	6.5E-05	0.00	0.00	0.06	0.009	4320	34

Table I. Parameters obtained by fitting model E (linear, CD95hi first) to labelling, telomere and YFV data. Table shows best fit estimates of the parameters and the corresponding standard errors (SE) obtained by the asymptotic covariance matrix method

Model ID	Model	SSQ DW19	SSQ DW20	SSQ DW25	Number of free params	AICc DW19	AICc DW20	AICc DW25
A	Indep homog.	109.5	72.2	85.1	4	51.9	15.0	11.4
B	Indep heterog.	74.4	67.0	77.3	7	9.0	13.3	6.6
C	fork	69.3	59.5	73.2	7	0.0	0.0	0.0
D	linear TN-> Tint->Thi	93.7	64.3	78.6	7	38.6	8.7	8.5
E	linear TN-> Thi->Tint	69.9	62.2	78.0	7	1.1	4.9	7.6

Table J. Summary of the fits of the 5 models to the labelling, telomere and YFV vaccination data. For each of the 5 models and the three individuals (DW19, DW20, DW25) the sum of squared residuals (SSQ) and the small sample corrected Akaike Information Criteria (AICc) is shown. As the absolute value of the AICc is meaningless, the AICc values have been normalised to the winning model (i.e. lowest AICc has been subtracted from all AICc). The winning model (the model with the lowest AICc) is highlighted in gold. Overall, the fork model (T_N->CD95int->CD95hi) performs best. Although the model fits for model A and B aren't too bad when assessed by the AICc we rule them out as the shape of the fit to the YFV data is totally unrealistic (both models predict no decline in the YFV-specific population).

Model ID	Model	SSQ DW19	SSQ DW20	SSQ DW25	Number of free params	AICc DW19	AICc DW20	AICc DW25
A	Indep homog.	45.1	11.8	26.1	3	146.7	68.5	33.4
B	Indep heterog.	15.6	6.2	18.8	6	17.7	2.3	0.9
C	fork	14.4	6.4	20.2	5	4.9	4.5	6.9
D	linear TN-> Tint->Thi	13.6	6.1	18.7	6	0.0	0.0	0.0
E	linear TN-> Thi->Tint	16.0	6.5	21.3	6	20.3	8.5	15.9

Table K. Summary of the fits of the 5 models to the labelling data only. For each of the 5 models and the three individuals (DW19, DW20, DW25) the sum of squared residuals (SSQ) and the small sample corrected Akaike Information Criteria (AICc) is shown. As the absolute value of the AICc is meaningless, the AICc values have been normalised to the wining model (i.e. lowest AICc has been subtracted from all AICc). The winning model (the model with the lowest AICc) is highlighted in gold. Overall, the linear model with CD95int first (T_N->CD95int->CD95hi) has the lowest AICc.

Model ID	Model	SSQ DW19	SSQ DW20	SSQ DW25	Number of free params	AICc DW19	AICc DW20	AICc DW25
A	Indep homog.	0.0667	0.0667	0.0667	4	12.9	21.9	24.5
B	Indep heterog.	0.0667	0.0667	0.0667	7	20	29	31.6
C	fork	0.0630	0.0609	0.0622	7	15.7	22.3	26.3
D	linear TN-> Tint->Thi	0.0509	0.0451	0.0436	7	0	0	0
E	linear TN-> Thi->Tint	0.0628	0.0608	0.0626	7	15.5	22.2	26.8

Table L. Summary of the comparison of the 5 model predictions with the unseen, unfitted YFV vaccination data. For each of the 5 models and the three individuals (DW19, DW20, DW25) the sum of squared residuals (SSQ) and the small sample corrected Akaike Information Criteria (AICc) is shown. In this case, as the models are not fitted to the data it makes no sense to penalise by the number of parameters and so the AICc will be unfairly low for model A (we just show the AICc as an easy metric for comparing goodness of fit). The winning model (the model with the lowest AICc) is highlighted in gold. Overall, the linear model (T_N->CD95int->CD95hi) has the lowest AICc. More convincing than the AICc is the plot of the predictions as the other two viable models from the previous stage (Model C and Model E) fundamentally fail to predict the CD95hi TSCM data (**Figures Q** and **R in S1 Text**). We conclude that, when testing its ability to predict an unseen data set, the linear model with Tint first performs considerably better than all other models.
