# Supplementary material for: Two distinct subpopulations of human stem-like memory T cells exhibit complementary roles in self-renewal and clonal longevity
Source: PLoS Biol. 2025 Jun 20;23(6):e3003179. doi: 10.1371/journal.pbio.3003179 (PMC12251145; doi:10.1371/journal.pbio.3003179)
Supplement: Attachment — Submitted filename: Response to Reviewers.docx [file pbio.3003179.s009.docx]

Changes (tracked) are shown in the marked-up version of the manuscript in red underline. In two places the changes were too extensive to track (it just caused a mess) so in this case we have indicated the new text in green font (page 8 and page 9). We have also added six new figures (Figure 11, Supplementary Figure 2A and 2C, Supplementary Figure 4, Supplementary Figure 8A and 8B) and moved one figure from the main text to the supplementary information (now Supplementary Figure 9). Again, these new figures are not tracked.

**Reviewer #1:**

This is a very thoughtful and interesting manuscript which uses flow cytometric, cell labelling, and molecular techniques to distinguish the activity of two human T memory stem cell subpopulations. The authors then generate and test five mathematical models linking the two subsets, and retest their analyses using a completely different set of cell surface markers on a different clinical T cell population from a published dataset. Overall, the work is very thoughtful and well-executed. In fact, I only have one experimental and two textual, requests for revision.

1) First, given their relative numbers over time, can the authors quantitate the approximate numbers of the two populations (in LN + Blood) over an individual's lifetime;

We can make a rough estimate of the total count of CD95int and CD95hi TSCM in LN and blood with age. First, we have the proportions of CD8+ T cells that are CD95int TSCM and CD95hi TSCM in blood:

|  |  |
| --- | --- |

Together with the proportions of CD8+ T cells that are CD95int TSCM and CD95hi TSCM in lymph node (the 95% confidence intervals given by the shaded area, are larger than the corresponding intervals in blood due to the smaller number of volunteers giving lymph node aspirates):

|  |  |
| --- | --- |

Then, to scale these proportions to counts in the whole body we use the following estimates for the blood scaling: the average CD8+ T cell count per microlitre is 800 cells and the blood volume of an average adult is 5L (1, 2). Together with these estimates for the LN scaling: 41.3% of body lymphocytes are found in the LN and tonsil, there are approximately 400-500x10^9^ lymphocytes in an adult and approximately 10% of LN lymphocytes are CD8+ T cells (3, 4). Since only a proportion of the blood and LN measurements are matched, we cannot simply sum the blood counts and LN counts measurement by measurement, instead we sum the trend lines. Giving the following final estimates for total count of CD95int TSCM (left) and CD95hi TSCM (right) in blood and LN with age:

|  |  |
| --- | --- |

2) I would invite the authors to speculate on how the intermediate and high populations function kinetically in vivo: i.e. does the intermediate population divide much more infrequently than the high population; (this would be similar to the recent claims about slightly more mature hematopoietic cells (MPPs) than HSCs demonstrating both self-renewal and ebullient proliferative differentiation;

We can, in fact, calculate how frequently the two subpopulations divide in vivo from the stable isotope labelling data. As you suggest, the less mature (CD95intermediate) population divides much less frequently than the CD95hi population. We estimate that on average a CD95intermediate cell divides once every 3.4 years whereas a CD95hi cell divides approximately once a year. This, together with the significantly increased self-renewal of the more mature CD95hi TSCM subpopulation makes for a very interesting parallel with the HSC/MPP system as you suggest. We have now added this information to the legend of the table of parameters (Table 1) and included a paragraph on this in the discussion.

3) I do think the authors should note that the first suggestion of T Memory Stem Cells in mice was published by Zhang, Emerson et al. in 2005 in Nature Medicine (in mice, Sca1 was a useful marker), just to be fair and accurate.

This is an important point. We have now amended the introduction to read “Although T_SCM_ cells were first described in mouse models of graft v host disease^(5)^, …” and have also edited the reference to Gattinoni et al to make clear that this work was in humans.

**Reviewer #2:**

This is an extensive study that makes the case for two distinct lineages within human stem-like memory T cells, distinguished by their levels of CD95 expression. The authors previously inferred heterogeneity within TSCM (del Amo Plos Biol 2018) but did not identify a marker differentiating the two subsets. This latest study combines an even wider array of assays and mathematical modelling tools - the force of the combined approach is impressive. It is established that CD95int cells divide and are lost at slow and almost-balanced rates, giving very long clonal lifespans. CD95hi cells are more proliferative but also die rapidly, giving shorter clonal lifespans. The relationship between the lineages is not definitively established but the data are consistent with a linear model in which the slow CD95int cells continually seed faster and shorter-lived CD95hi cells.

it wasn't totally clear to me why the two populations are collectively stem-like (abstract). If one thinks of clonal persistence it seems that the CD95int cells really are the stem-like candidate.

Our argument is that clonal persistence isn’t the only necessary attribute of a stem cell population. We argue that stem cells have three key properties that are essential if a population is to function as a stem cell population -these are self-renewal (it has to be able to maintain itself upon proliferation to expand if necessary), clonal longevity (proliferation and death need to be closely matched to enable long lived immune memory) and multipotency (so that all the necessary downstream populations: central memory , effectors, effector memory can be produced upon meeting cognate antigen again). CD95int TSCM show multipotency and clonal longevity whilst CD95hi TSCM show multipotency and self-renewal. So together CD95int and CD95hi TSCM have all three properties of a stem cell population.

It would be good to discuss further the potential drivers of int->hi conversion. It is modelled as constitutive but could this be an intermittent, antigen-driven process? If it's recruitment from naive cells does this mean, in the case of YFV, that

The end of your question was cut off in the email we received from PLOS but we attempt to answer what we think your question is. We think constitutive antigen-independent conversion of int->hi is probable as otherwise, due to the relatively short half life of the CD95hi subpopulation, this CD95hi population would go extinct more quickly than observed (if it wasnt been replaced by the long-lived upstream CD95int population). Below we plot this for the YFV-specific CD95int and CD95hi populations (black dots are the cross sectional data as discussed in the manuscript, coloured lines are model predictions of the winning model (model D) not fits, generated using our parameter estimates (Table 1) but blocking flow from CD95int to CD95hi (i.e. setting \Delta_1=0).

You can see we capture the dynamics of the CD95int population over time but for DW19 (red line) we tend to underestimate the CD95hi population if we do not allow CD95int->hi conversion after the initial acute infection is cleared (DW20, green, shows the same problem to a lesser extent). I think this argues for ongoing conversion from CD95int->hi. Given that there is no evidence for YFV persisting for years after vaccination (either as intact virus or as antigen) then I think this argues for ongoing conversion of CD95int to hi in the absence of antigen at least for one (maybe two) of the three individuals.

The 7 day assays looking at outcomes of IL15 and IL2 stimulation are really interesting in this regard. Could you use that information to help speculate how these TSCM subsets are related and embedded within memory subsets as a whole? They are in vitro assays but they do have some directionality information in there.

You are right the in vitro culture work is informative and we probably don’t give another attention to it. I don’t know if you’ve seen Supp Figure 3? That is particularly interesting for understanding the connections between the TSCM subpopulations and the wider memory pool. The results are slightly different for Il-2 and Il-15 but broadly there seems to be a pattern where CD95int give rise to CD95hi (and Temra) but that CD95hi don’t really give rise to CD95int (consistent with the lineage directionality implied by our other data -labelling, TRECs, gene expression). Looking at the wider memory populations both TSCM subpopulations appear to give rise to TEMRA more readily than they give rise to TCM or TEM which (to me at least) is unexpected though I would want to repeat the experiment with more markers or with RNAseq to be convinced that these were genuine TEMRAs. TCMs on the other hand (and in marked contrast to their response to TCR stimulation, Figure 2C) give rise to a broad profile of cells including TCM, TEM and TEMRA (as well as CD95hi TSCM). We don’t want to overinterpret these results as the in vitro system can never fully recapitulate the in vivo one but we have summarised these finding in a couple of places:

“In response to αCD3αCD28 stimulation, a strikingly different pattern emerged. In this scenario, both TSCM subpopulations rapidly differentiated whereas a large proportion of divided TCM cells retained their phenotype (Figure 2B). Investigating this further, we found that both TSCM subpopulations were able to differentiate into a number of other phenotypes: TCM, TEM and TEMRA were all generated by both CD95hi and CD95int TSCM cells in appreciable quantities, Figure 2C. In contrast TCM were only able to produce TEM in measurable quantities, Figure 2C. Quantifying this as a “multipotency index” (Methods), we found that both CD95hi TSCM and CD95int TSCM had a significantly higher multipotency index than TCM (P=0.01, N=4 and P=0.0004 N=7 respectively, unpaired Wilcoxon rank sum, Figure 2D).

Together, these results suggests that CD95hi TSCM are both better able to maintain themselves during homeostasis but also better able to generate a multicellular immune response during challenge when compared with TCM cells. ” (Results)

And

“This [linear model whereby CD95 int give rise to CD95hi] is further supported by the in vitro differentiation data whereby CD95int TSCM give rise to CD95hi TSCM but CD95hi TSCM tend not to give rise to CD95int TSCM (Supplementary Figure 3).” (Discussion)

it would be good to quote cell lifespans as well as clonal half-lives. Alternatively, you could combine death and differentiation to define a mean residence time 1/(delta + Delta). I think timescales are a bit easier for everyone to digest than rate constants. A related parameter is then Delta/(Delta + d) which is the proportion cells lost from CD95int population that seeds the CD95hi subset - a sort of conversion efficiency. Maybe a figure with all of these relationships and rates?

It’s a good idea to include a figure summarising all the timescales, we have now added this to the revised manuscript (Figure 11).

The topology question is very interesting. The authors use goodness-of-fit criteria to establish support. As is also often the case, the fits of the different models are visually quite similar so we are putting a lot of trust in the statistics. The AIC values presumably include residuals from multiple datasets - are these weighted in any way? It's usual to normalise each set of squared residuals by the variance of the observations in that dataset - those variances are actually free parameters, though not usually estimated with simple least squares performed with one dataset, where you can use the AICc formula with the SSR directly. It becomes more important when combining multiple datasets with different levels of uncertainty in the observations. With least squares fitting you can skirt around this by plugging in the ML estimate of each dataset's variance (see Hogan PNAS 2015) - this essentially gives you a set of weights for the SSRs from different datasets (there are other more rigorous ways to do it in Bayesian frameworks).

I don't want to labour this point as it's a bit technical but for me the relationship between these two subsets is the most intriguing aspect of the paper and so it would be great to see more of the methodology.

Regarding the issue of visually similar fits and the required trust in the statistics, we fully take your point. We have therefore rewritten the manuscript removing the emphasis on the AICc for model choice. Instead, we only rule out models if their fit is catastrophic and fails to fit a basic feature of the data. This means that after fitting the labelling, YFV and telomere data then three models rather than one are now viable (fork and the two linear models) -the two independent models fail completely as they do not predict the decline in YFV-specific cells (see Supplementary Figures 2C and 3C). Upon comparing predictions (not fits) of these three viable models to the unseen YFV TSCM CD95int and hi data set then the fork model and the linear Thi model can be ruled out as they predict a very rapid loss of the CD95hi population that is fundamentally incompatible with the data (Supp Fig 17 and 18). This leaves us with the linear model (Tint first) as the winning model. As these changes required quite a lot of edits the changes are not tracked -it caused chaos – the edited paragraphs are the paragraph starting “We constructed” on pg 8, and text from “We simply predicted” to the end of that paragraph on pg 9 (both highlighted with green font in the tracked changes manuscript). Figure 10 (now 9) has also been edited and two new figures (Supp Fig 17 and 18) added.

**Reviewer #3:**

Background: The work by Koftori, Asquith and colleagues builds upon seminal work by Gattinoni et al 2011 who identified a novel T cell subset with stem cell like properties, defined as naïve phenotype and CD95+. This stem cell memory populated had the ability to self-renew and repopulate the memory subsets. An important follow-up study by Costa del Amo et al 2018 studied the dynamics of stem cell memory revealing that stem cell memory are likely composed of two subsets. One subset has properties of a long-lived cell while the other is short lived.

Current work: In the current study Koftori demonstrates the stem cell memory can be divided into two subpopulations: those that express high levels of CD95 (CD95hi) and those that express intermediate levels of CD95 (CD95int). They show these two populations are distinct in many ways including (1) their half-lives and (2) their ability to self-renew. Specifically, the CD95hi cells had a much shorter half-life and a greater ability to self-renew and were more similar to CM while the CD95int shared characteristics with naïve T cells. Finally the authors utilize a modeling approach to suggest that naïve T cells give rise to CD95int which give rise to CD95hi which give rise to CM. This model builds on several sources of data and is consistent with the data in the figures as well. Moreover, the model invites new avenues of research.

Context: Notably, other group have subdivided stem cell memory into subpopulations based on different surface markers. In particular, Zhao et al 2021 subdivided TSCM into CD122 hi and CD122 low. However, this prior study using CD122 was less convincing. Specifically, the sorted populations did not convincingly separate the ability to self-renew; nor did they even measure the half-life. This is an important omission since it is the entire basis for the hypothesis that there were two subsets within the stem cell memory population.

Data and analysis: Almost all of the data that would be needed to replicate the study are available, except for Figures 2-4, comment 2-4. The statistical analysis is rigorous and robust.

Major Comments:

1.	It appears the authors have been able to separate the frequently dividing cells from the slow dividing subset better than prior studies. However, the data should be more comparable to the prior study by Zhao et al. 2021 so that the reader would be more informed when choosing markers to sort separate the stem cell memory population. In particular, the authors should sort 4 populations (CD95hi+CD122hi; CD95hi+CD122lo; CD95int+CD122hi; CD95int+CD122lo and repeat the experiments in Figure 2A and 2C.)

This is an interesting idea which we attempted. However, we encountered problems of low cell count in the 4 sorted subpopulations. We anticipated in advance that cell numbers would be low with such tightly defined subpopulations so we tried sorting cells from both 100ml blood from healthy donors as well as from donor cones nevertheless the numbers of sorted cells were low:

|  | Total cells sorted from 100ml of blood/Donor Cones | | | | | |
| --- | --- | --- | --- | --- | --- | --- |
|  | Donors | | | | | |
| **Populations** | **115** | **116** | **117** | **120** | **DC1*** | **DC2*** |
| **CD95intCD122hi** | 13018 | 15770 | 15000 | 9707 | 15199 | 21238 |
| **CD95intCD122lo** | 8918 | 18900 | 18600 | 8433 | 5599 | 12727 |
| **CD95hiCD122lo** | 18890 | 9207 | 17609 | 7805 | 13133 | 26650 |
| **CD95hiCD122hi** | 18597 | 4895 | 7000 | 3587 | 29860 | 19409 |

* donor cones

We only had enough cells for one culture condition so we decided to prioritise the Il-7/Il-15 condition. However, we found poor viability with all of the cell populations of interest having a median viability of less than 40%. In contrast, the naïve population had a median viability of greater than 70%, furthermore we had not previously had issues of viability when working with the (larger) CD95int and CD95hi subpopulations suggesting that the problem might have been associated with sorting such small subpopulations (see figure below).

|  | **Figure.** Viability of the sorted subpopulations after 7 days *ex vivo* culture. |
| --- | --- |

We went ahead and looked at the proportion of divided cells for the different subpopulations (graph below) but with such low cell viability it is difficult to trust these results. Furthermore, the low number of events in the CTV-diluted gate means that the estimates of self-renewal are unlikely to be reliable and so we were not able to take this any further forward.

|  | **Figure.** The proportion of divided cells (by CTV staining) in the 4 subpopulations of interest together with naïve cells as a control. |
| --- | --- |

2.	Figure 2: The authors should show the flow histogram analysis of their CellTraceViolet along with the proliferation index (akin to Zhao et al Eur. J. Immunol 2021) for CD95 hi and CD95int. Again this would make their study more comparable to the Zhao study.

This is a good idea and we have now included the requested figure (Supplementary Figure 4 in the revised manuscript).

3.	Supplementary figure 2 provides gating strategy. This gating strategy does not include the live/dead staining nor the cell trace staining. The authors should provide the full gating strategy starting with FSC vs SSC.

We have now updated Supplementary Figure 2A to show the full gating strategy starting from FSC/SSC. We have also added a new panel (Supplementary Figure 2C) showing CTV staining (together with Supplementary Figure 4 which shows CTV profiles following division).

4.	Please provide heat maps of the most variable genes (akin to Zhao 2021) to show the differentially expressed genes in the different subsets with and without stimulation. It is more intuitive to see similarities between subsets with the heat map.

We have now included the heat maps of gene expression with and without stimulation as requested (Supplementary Figures 8A and 8B in the revised manuscript).

5.	Figure 3 A and B(qPCR) comparing fold change gene expression in CD95int/CD95hi) is not very convincing to me. The authors could delete it. The data tables while transparent leave too much work for the reader/reviewer. If the authors prefer to keep this data, they should limit the analysis to genes that were actually differentially expressed between the critical subsets in their data set and remove gene sets that were not detected in any samples.

We have moved Fig 3A & B as requested. We have instead included them as Supplementary Figures (now Supplementary Figures 9); we think it is a useful validation of the RNAseq work showing that CD95int TSCM are closer to naïve whilst CD95hi TSCM are closer to CM. We have also deleted the table of PCR results from the Supplementary Tables, we agree it was very large and difficult to read. PLOS Biology have asked us to make this data available though and as there is no dedicated repository for qPCR data we have uploaded it as a supplementary file (Supp File1.xlsx) so others can check our results and reuse the data if they wish.

Minor Comments:

1.	Figure 2 would benefit from unique identifiers.

We recreated Figure 2 with unique identifiers (see example plots below for Fig 2A and 2B) but I’m not sure that is useful so I have left the original in the manuscript for now. If you think it is useful let us know and we can add it in.

**A. Self-renewal**

**B. Response to TCR stim**

2.	The sentence "The only T cell pathway (CSE21360, PRIMARY vs QUATERNARY MEMORY CD8 T CELL UP from 19) implies that genes upregulated in CD95hi Tscm are enriched in genes upregulated in memory CD8 T cells following multiple antigen challenge whereas genes upregulated in CD95int are associated with memory CD8 T cells following primary antigen challenge" should be further clarified.

We have now written:

Only one of the enriched pathways was a T cell pathway (GSE21360, PRIMARY VS QUATERNARY MEMORY CD8 TCELL UP from ^20^). This suggests that genes upregulated in CD95hi TSCM are enriched in genes upregulated in memory CD8 T cells following multiple antigen challenge (“QUATERNARY MEMORY CD8 TCELL”) whereas genes upregulated in CD95int TSCM are associated with memory CD8+ T cells following primary antigen challenge (“PRIMARY MEMORY CD8 TCELL”).

If you have further suggestions on how to clarify this further, then we’d be very happy to incorporate those.

3.	Please fix the y axis label in Figure 7b

Thank you for pointing this out. The y axis in Figure 7 (now Figure 6) has been corrected.

4.	The telomere analysis from a published dataset could be deleted as it is not needed to make the conclusions of the story.

We prefer to leave the telomere data in as we are showing the goodness of fit of the model to the telomere data and this is important for distinguishing between the different models and estimating the model parameters.

The additional work requested by Reviewer 3 was performed by Charandeep Kaur and Laura-Mora Bitria. Following discussions with Laura, Charan and the first author (Danai Koftori) we agreed that as the additional work was quite laborious, and they had already contributed considerably to the original submitted version of the manuscript, it would be fair to reflect their contribution by starring them as joint first authors with Danai. This change has now been made in the revised manuscript.

References cited in response to reviewers

1.	University of Rochester, Medical Encylopedia [Available from: <https://www.urmc.rochester.edu/encyclopedia/content?contenttypeid=167&contentid=cd4_cd8_ratio>.

2.	NHS Blood and Transplant [Available from: <https://www.blood.co.uk/news-and-campaigns/the-donor/latest-stories/blood-group-basics/>.

3.	Trepel F. Number and distribution of lymphocytes in man. A critical analysis. Klin Wochenschr. 1974;52(11):511-5.

4.	Battaglia A, Ferrandina G, Buzzonetti A, Malinconico P, Legge F, Salutari V, et al. Lymphocyte populations in human lymph nodes. Alterations in CD4+ CD25+ T regulatory cell phenotype and T-cell receptor Vbeta repertoire. Immunology. 2003;110(3):304-12.

5.	Zhang Y, Joe G, Hexner E, Zhu J, Emerson SG. Host-reactive CD8+ memory stem cells in graft-versus-host disease. Nat Med. 2005;11(12):1299-305.
