# Supplementary material for: Two distinct subpopulations of human stem-like memory T cells exhibit complementary roles in self-renewal and clonal longevity
Source: PLoS Biol. 2025 Jun 20;23(6):e3003179. doi: 10.1371/journal.pbio.3003179 (PMC12251145; doi:10.1371/journal.pbio.3003179)
Supplement: Attachment — Submitted filename: response_to_reviewers_auresp_3.docx [file pbio.3003179.s010.docx]

------------------------------------------------------------------------

REVIEWER'S COMMENTS:

------------------------------------------------------------------------

Reviewer #3:

We thank the authors for their thoughtful responses to our comments and would like to accept the manuscript. Overall, it is an important contribution and a large body of work and the reviewers were responsive to all of my queries. It is unfortunate that sorting the cells into CD95hi/int CD122hi/int yielded low cell numbers, but we appreciate the effort.

Thank you very much for all your suggestions which have helped to improve our manuscript.

One minor note to discuss is the authors mention in their abstract and manuscript that the CD95hi cells have considerably higher proliferation than the CD95int cells. The in vivo data in Figure 6 supports their claim, however there is inter-donor variability. The authors could discuss the variability in their data which could easily related to variable after cell sorting.

This is an important point. We have now included a discussion on this point in the revised version of the manuscript (pg 14, paragraph marked in blue font).

I appreciate the addition of the RNA heat maps in Supplemental Figure 8. Could the authors move LD2_int_U with the other intermediate cells in Supplemental Figure 8a.

The clustering of the cell populations in the heatmaps is unsupervised (Methods). It would not be appropriate to move the population manually (this could be construed as data manipulation).

I prefer the unique identifiers in figure 2 as it helps the reader understand donor variability.

We have now included the figure with unique identifiers as Supplementary Figure 19.
